# Role of Oral–Lung Infection Axis on Respiratory Health

**DOI:** 10.3390/biomedicines14081817

**Published:** 2026-08-13

**Authors:** Ozge Unlu, Mehmet Demirci, Alpdogan Kantarci

**Affiliations:** 1 Department of Medical Microbiology, Faculty of Medicine, Istanbul Atlas University, 34408 Istanbul, Türkiye; 2 Department of Medical Microbiology, Faculty of Medicine, Kırklareli University, 39100 Kirklareli, Türkiye; mehmetdemirci@klu.edu.tr; 3 Department of Developmental and Surgical Sciences, School of Dentistry, University of Minnesota, Minneapolis, MN 55455, USA; kantarci@umn.edu; 4 School of Dental Medicine, Harvard University, Boston, MA 02115, USA

**Keywords:** oral–lung axis, oral microbiome, dysbiosis, chronic obstructive pulmonary disease (COPD), periodontitis, *Porphyromonas gingivalis*, mucosal immunity, micro-aspiration, biofilm interactome, nano-theranostics

## Abstract

High-throughput metagenomic sequencing and advances in mucosal immunology have refuted the traditional physiological concept of a sterile respiratory tract. The oral cavity has been recognized as a dynamic determinant of systemic health. As in other parts of the body, recent studies also suggest that pulmonary health may be linked to oral health. Under eubiotic conditions, the oral microbiome maintains local immunological homeostasis and colonization resistance. Oral dysbiosis, characterized by sequential shifts in microbial communities and the proliferation of the pathogenic red complex (*Porphyromonas gingivalis*, *Treponema denticola*, and *Tannerella forsythia*), induces a state of chronic systemic inflammation, potentially involved in an infectious axis between the oral cavity and the lung. This review evaluates the tripartite systemic pathways of metastatic infection, metastatic injury, and metastatic inflammation that govern the translocation of oral pathobionts and their bioactive components, including lipopolysaccharides, outer membrane vesicles, and matrix metalloproteinases, to the lower respiratory tract via microaspiration and hematogenous circulation. The clinical implications across the chronic respiratory disease spectrum are examined, with a focus on how deficits in oral microbial diversity influence chronic obstructive pulmonary disease (COPD) pathogenesis, modulate the pulmonary virome and mycobiome, and stimulate maladaptive trained immunity. Furthermore, the contribution of biological aging is assessed, highlighting the roles of immunosenescence, inflammaging, and physiological reflex decline within the broader mucosal continuum. Finally, the clinical translation of this axis is analyzed, emphasizing the integration of saliva-based point-of-care nano-theranostics, metatranscriptomic profiling, and targeted interventions—such as professional oral biofilm management in intensive care settings and precision microbiome engineering—to preserve respiratory function and restore immune homeostasis.

## 1. Introduction

The human oral cavity represents one of the most anatomically complex and ecologically dynamic interfaces between the host and the external environment, housing a highly specialized mucosal immune network [1]. Traditional physiological theories reduced the mouth to a mechanical conduit, a passive portal responsible only for the initial conduction of air and the intake of nutrients. The advent of high-throughput metagenomic sequencing and advanced mucosal immunology has challenged this reductive view, positioning the oral cavity as a key determinant of systemic health [2,3]. The oral environment, far from being a separate anatomical compartment, sustains a complex symbiotic equilibrium that has significant, far-reaching regulatory impacts on extra-oral organ systems [1,2,3,4]. Epidemiological, clinical, and translational lines of evidence have linked periodontal diseases and oral dysbiosis to more than 50 systemic conditions, including cardiovascular diseases, type 2 diabetes, adverse pregnancy outcomes, and neurodegenerative disorders [5,6,7].

Among the various routes of systemic translocation, the oral–gut axis has historically been the most extensively investigated, demonstrating how swallowed oral pathobionts survive the gastric acid barrier and colonize the lower gastrointestinal tract, thereby disrupting intestinal homeostasis [8]. This framework was later expanded into the sophisticated “oral–gut–brain axis,” illustrating how gut-derived systemic inflammation and metabolic byproducts cross the blood–brain barrier to modulate neuroinflammation and cognitive functions [9,10]. These paradigms have prompted researchers to hypothesize that similar networks exist elsewhere in the body, leading to growing interest in alternative mucosal frontiers. Within this context, the “oral–lung axis” has emerged as a compelling and biologically plausible concept [11,12]. Given the direct anatomical continuity, shared aerodigestive tract, and continuous aerodynamic exchange, the oral–lung axis is now recognized as a critical, yet historically underappreciated, determinant of pulmonary resilience and respiratory health [12,13].

### Literature Search Strategy

To ensure a comprehensive and up-to-date integration of scientific literature, a structured literature search was conducted across PubMed/MEDLINE, Scopus, Web of Science, and Google Scholar databases for peer-reviewed studies published up to 2026. The search strategy employed combinations of Medical Subject Headings (MeSH) terms and free-text keywords, including: (“oral–lung axis” OR “oral microbiome” OR “periodontitis” OR “*Porphyromonas gingivalis*”) AND (“chronic obstructive pulmonary disease” OR “COPD” OR “pneumonia” OR “micro-aspiration” OR “mucosal immunity” OR “metatranscriptomics” OR “trained immunity”). Studies were selected based on their relevance to oral microbial dysbiosis, systemic translocation pathways, respiratory disease pathogenesis, and clinical translational applications. Priority was given to high-impact original research articles, randomized controlled trials, large-scale epidemiological cohorts, Mendelian randomization studies, and mechanistic in vivo/in vitro investigations. Reference lists of relevant articles were also manually screened to identify additional eligible studies.

## 2. The Oral Landscape: A Reservoir of Diversity

The oral cavity harbors a remarkably diverse and dense microbial consortium, second only to the gastrointestinal tract, comprising over 700 prokaryotic species organized into complex biofilm communities across distinct ecological niches [14,15]. In a state of eubiosis, this landscape is characterized by high taxonomic diversity and functional redundancy. The healthy oral microbiome is predominantly dominated by commensal phyla such as *Firmicutes* (chiefly *Streptococcus* species like *S. mitis*, *S. oralis*, and *S. salivarius)*, *Actinomycota* (*Actinomyces* spp.), and *Proteobacteria (Neisseria* and *Eikenella* spp.) [16]. The symbiotic communities actively contribute to host defense through several well-characterized mechanisms [17]. They physically occupy cellular receptors to prevent the attachment of exogenous pathogens, synthesize antimicrobial peptides known as bacteriocins, and maintain a local biochemical equilibrium. For instance, the reduction in dietary nitrate to nitrite by commensal *Veillonella* and *Actinomyces* species supports systemic vascular tone and endothelial health via the nitrate-nitrite-nitric oxide pathway [18]. This stable homeostatic balance regulates the local baseline immunological tone, educates host resident immune cells, and ensures that mucosal defenses remain vigilant yet non-destructive along the shared aerodigestive pathway [19].

A shift from eubiosis to dysbiosis, driven by poor oral hygiene, smoking, or host immune deficiencies, alters ecological pressures in the oral cavity, favoring the overgrowth of pathobionts [20,21]. To understand the structural evolution of this dysbiotic shift, one must recall the pioneering framework established by Sigmund Socransky and colleagues in 1998. Socransky categorized oral bacteria into specific microbial complexes based on their pathogenicity, metabolic interdependencies, and sequential colonization patterns within subgingival biofilms [22]. The early colonization phase is dominated by the relatively benign Blue, Yellow, Purple, and Green complexes, which modify the local environment and pave the way for the Orange complex, which includes the bridging species. The Orange complex, heavily represented by *Fusobacterium nucleatum*, acts as a critical physical and metabolic bridge [22,23]. Due to its unique co-aggregation capabilities, *F. nucleatum* expresses surface adhesins that bind to both early commensals and late obligate anaerobes, facilitating the recruitment and structural stabilization of the highly pathogenic Red Complex [24].

The Red Complex, comprising *Porphyromonas gingivalis*, *Treponema denticola*, and *Tannerella forsythia*, constitutes the most virulent periodontal consortium [22,25]. Once established, these keystone pathogens substantially disrupt the host immune response [26]. Rather than directly causing mass destruction, they remodel the local microenvironment by disabling leukocyte killing mechanisms, thereby turning the periodontal pocket into a hyperpermeable, chronic “inflammation factory” [1,27]. Recent breakthroughs in targeted antimicrobials have demonstrated that the selective elimination of *F. nucleatum* disrupts this sequential assembly, breaks the metabolic bridge, alleviates periodontal tissue destruction, and dampens the local inflammatory cascade [28]. When left unchecked, the chronic tissue destruction mediated by the Red Complex leads to continuous leakage of bacterial lipopolysaccharides (LPS), outer membrane vesicles (OMVs), and pro-inflammatory mediators into the systemic circulation [29]. This sustained systemic stimulation has been closely implicated in a broad spectrum of extra-oral pathologies, modulating the systemic inflammatory tone [30]. Specifically, oral microbial dysbiosis has been directly identified as a prominent signature in patients with oral cavity cancers, demonstrating how chronic microenvironment perturbation can drive oncogenic pathways [31,32].

The systemic reach of this oral “inflammation factory” is highlighted by its distinct microbial signatures and links to altered immune tone in endocrine conditions such as euthyroid Hashimoto’s thyroiditis [33], as well as its profound correlation with systemic oxidative stress and subgingival alterations in patients with psoriasis [34]. Additionally, the presence of specific periodontal pathogens and local dysbiosis has been shown to strongly correlate with the severity of chronic systemic manifestations in patients with rheumatoid arthritis [35]. Taken together, Socransky’s sequential dysbiosis paradigm explains how a localized shift in the oral biofilm creates a powerful, continuous generator of systemic mediators capable of modifying distant mucosal frontiers, notably predisposed pulmonary tissues [36].

### Oral Dysbiosis Beyond the Lungs: A Brief Primer on Systemic Inflammatory Crosstalk

While the primary focus of this review centers on the oral–lung axis, the systemic impact of oral dysbiosis extends across multiple extra-pulmonary organ systems. Periodontal inflammation operates as a continuous systemic “inflammation factory,” where the ulcerated pocket epithelium allows the chronic leakage of lipopolysaccharides (LPS), outer membrane vesicles (OMVs), and pro-inflammatory cytokines into the circulation. This sustained low-grade systemic inflammatory state modifies host immune tone, contributing to the pathogenesis of diverse extra-oral conditions, including rheumatoid arthritis, euthyroid Hashimoto’s thyroiditis, psoriasis, and adverse pregnancy outcomes (such as preeclampsia and preterm birth). Rather than operating in isolation, these systemic comorbidities reflect a shared vulnerability driven by oral microbial translocation and unresolved systemic inflammation, which collectively lower the pathological threshold for chronic mucosal and distant tissue damage, including within the respiratory tract.

## 3. The Migration: How Mouth Microbes Reach the Lungs

The dissemination of oral microflora and their bioactive components to extra-oral sites is dictated by a tripartite pathological framework: metastatic infection, metastatic injury, and metastatic inflammation [37]. Understanding these fundamental systemic pathways is crucial before examining how oral dysbiosis specifically alters pulmonary homeostasis through a tripartite systemic pathway.

### 3.1. Metastatic Infection

Metastatic infection occurs when viable oral microorganisms breach the local oral vascular barrier and enter the systemic circulation, a phenomenon clinically recognized as transient bacteremia. Routine activities such as mastication and toothbrushing, as well as invasive procedures such as dental extractions, disrupt the hyperpermeable, ulcerated pocket epithelium in patients with periodontitis, thereby permitting the direct entry of bacteria into the bloodstream [38,39]. Once in the circulation, these pathobionts can escape host immune surveillance and colonize susceptible distant tissues. The classic archetype of this mechanism is infective endocarditis, in which oral viridans streptococci or HACEK organisms adhere to damaged endocardial surfaces or prosthetic valves, initiating biofilm formation and focal endovascular infection [40,41].

### 3.2. Metastatic Injury

Metastatic injury refers to the systemic dissemination of bacterial virulence factors and metabolic byproducts rather than the viable cells themselves [33]. Periodontal pathogens synthesize an array of noxious products, including cytolytic enzymes, exotoxins, and shed lipopolysaccharides (LPS/endotoxins) [29,42]. In chronic periodontitis, these biomolecules continuously enter the cardiovascular system, where they act as potent pathogen-associated molecular patterns (PAMPs). For instance, circulating *P. gingivalis* LPS directly activates endothelial Toll-like receptors (TLR-2 and TLR-4), inducing endothelial dysfunction, promoting the expression of cell adhesion molecules (ICAM-1, VCAM-1), and accelerating atherogenesis far from the oral cavity [43,44].

### 3.3. Metastatic Inflammation

**Figure 1:**
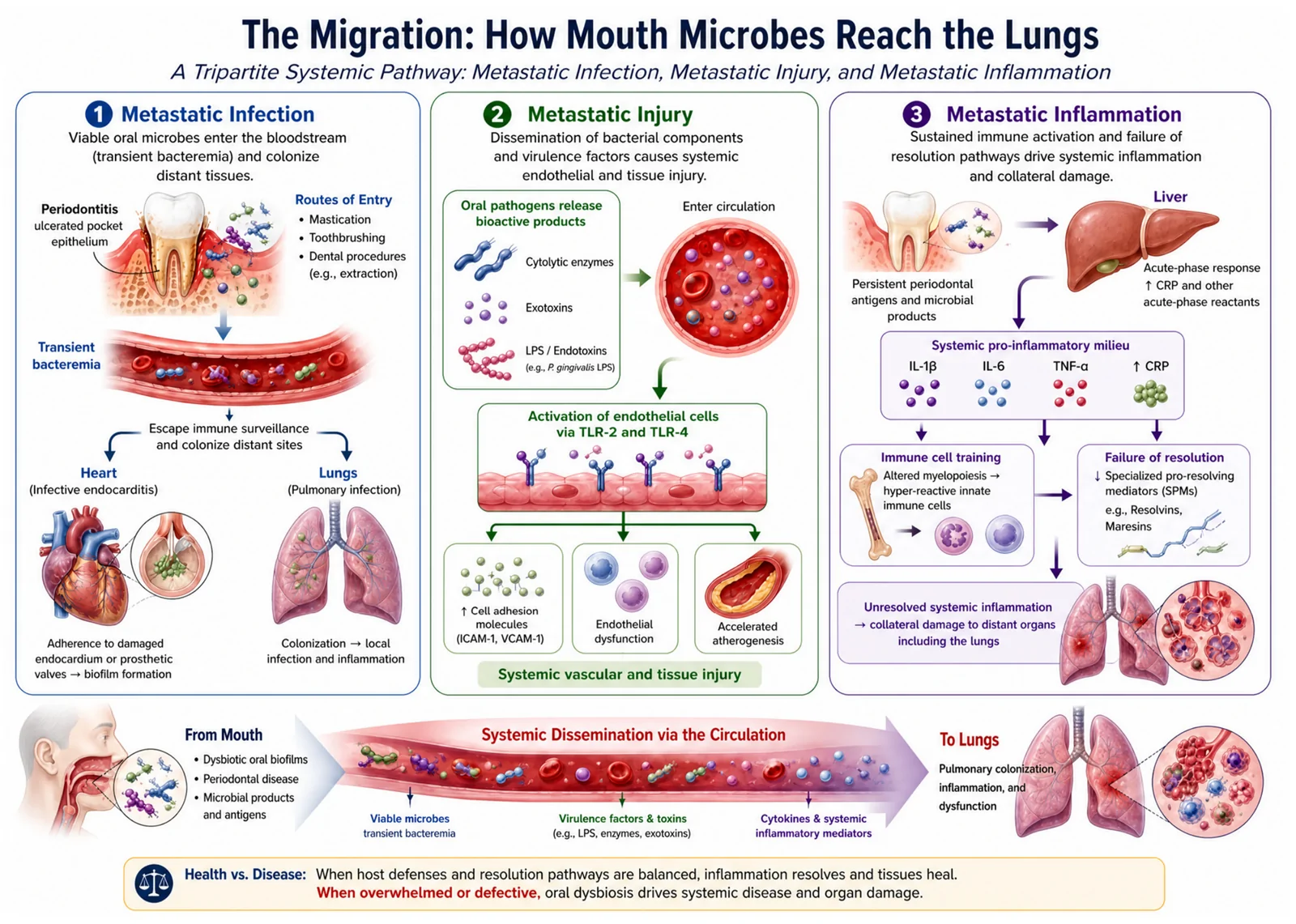
Tripartite systemic dissemination pathways linking oral dysbiosis to distant organ injury and pulmonary pathology. Panel 1 (Metastatic Infection): Illustrates how routine mechanical activities (mastication, toothbrushing) or invasive dental procedures disrupt hyperpermeable ulcerated pocket epithelium in periodontitis, allowing viable oral pathobionts to enter systemic circulation (transient bacteremia), escape host surveillance, and colonize distant tissues (e.g., endocardium or lungs). Panel 2 (Metastatic Injury): Depicts the systemic dissemination of bacterial virulence factors and shed lipopolysaccharides (LPS), which activate systemic vascular endothelial cells via Toll-like receptors (TLR-2 and TLR-4), upregulating cell adhesion molecules (ICAM-1, VCAM-1) and driving endothelial dysfunction. Panel 3 (Metastatic Inflammation): Shows sustained systemic immunological responses triggered by circulating antigens, leading to elevated hepatic acute-phase reactants (CRP), pro-inflammatory cytokines (IL-1β, IL-6, TNF-α), altered bone marrow myelopoiesis (trained immunity), and resolution failure due to specialized pro-resolving mediator (SPM) deficiencies. Abbreviations: CRP: C-reactive protein; ICAM-1: Intercellular Adhesion Molecule 1; IL-1β: Interleukin 1 beta; IL-6: Interleukin 6; LPS: Lipopolysaccharide; SPM: Specialized Pro-resolving Mediator; TLR: Toll-Like Receptor; TNF-α: Tumor Necrosis Factor alpha; VCAM-1: Vascular Cell Adhesion Molecule 1.

Metastatic inflammation is driven by the sustained, systemic immunological response to oral pathobionts and their antigens [7]. The persistent presentation of periodontal antigens triggers a chronic systemic inflammatory echo, marked by the hepatic upregulation and release of acute-phase reactants such as C-reactive protein (CRP) and the systemic elevation of pro-inflammatory cytokines (IL-1β, IL-6, and TNF-α) [45]. This systemic hyper-inflammatory state alters bone marrow myelopoiesis, training innate immune cells to adopt a hyper-reactive phenotype [7]. Crucially, the pathogenesis of this metastatic inflammation is not merely defined by the overproduction of pro-inflammatory mediators, but by a fundamental failure in the host’s endogenous resolution pathways [46]. Specialized pro-resolving mediators (SPMs), such as Resolvins and Maresins, are essential for actively halting leukocyte infiltration and promoting tissue homeostasis [47]. When these resolution pathways are overwhelmed or defective due to persistent oral dysbiosis, unresolved systemic inflammation can induce collateral damage in distant organ systems, lowering the pathological threshold for chronic destructive conditions [44,48]. The migration strategies of oral microorganisms to the lungs were outlined in Figure 1.

### 3.4. Pulmonary-Specific Translocation Routes

Micro-aspiration: Physiological micro-aspiration of oropharyngeal secretions during sleep occurs in a significant proportion of healthy individuals, but the aspirated fluids are efficiently cleared by the pulmonary mucociliary escalator and resident alveolar macrophages [49]. However, in patients with chronic periodontitis, the aspirated fluid is qualitatively transformed into a toxic inoculum, dense with virulent Red and Orange complex pathobionts, bacterial proteases, and host-derived salivary cytokines [50]. This chronic, high-load micro-aspiration represents a direct form of metastatic infection and injury combined. The aspirated oral pathobionts alter the local lung microenvironment, neutralizing the commensal lung microbiota, degrading the protective alveolar epithelial barrier, and overloading alveolar macrophages’ phagocytic capacity, thereby predisposing the lower respiratory tract to acute and chronic infectious challenges [2,51].

**Figure 2:**
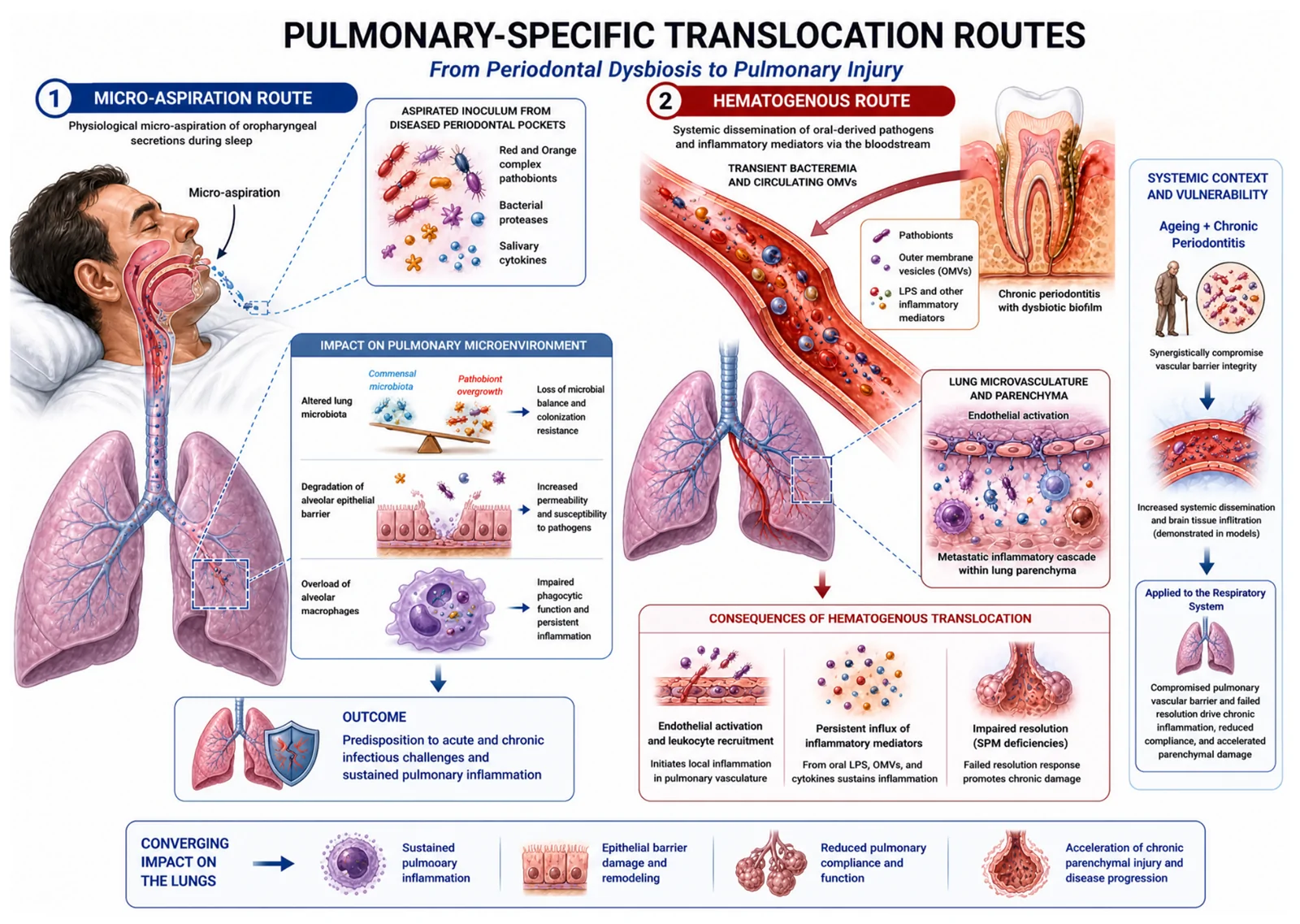
Pulmonary-specific translocation routes of oral pathobionts and their metabolites. Panel 1 (Micro-aspiration Route): Illustrates the direct anatomical passage of toxic oral inocula—rich in Red and Orange complex pathobionts, proteases, and salivary cytokines—into lower airways during sleep. This micro-aspiration alters lung microbiota balance, neutralizes protective commensals, degrades the alveolar epithelial barrier, and overloads alveolar macrophage phagocytic capacity. Panel 2 (Hematogenous Route): Demonstrates systemic dissemination via the pulmonary microvasculature, which receives total cardiac output. Circulating pathobionts and OMVs lock onto pulmonary endothelial surfaces, driving local inflammation, vascular barrier breakdown, and parenchymal tissue remodeling, which is synergistically amplified by biological aging and chronic periodontitis. Abbreviations: LPS: Lipopolysaccharide; OMV: Outer Membrane Vesicle; SPM: Specialized Pro-resolving Mediator.

The Hematogenous Route: Complementing micro-aspiration, the hematogenous route provides a continuous source of systemic inflammatory stimuli to the lungs. The lungs receive the entire cardiac output, making the pulmonary capillary bed uniquely exposed to the transient bacteremias and circulating OMVs originating from diseased periodontal tissues [52]. Circulating oral pathobionts and shed LPS lock into the pulmonary microvasculature, initiating local endothelial activation and a metastatic inflammatory cascade within the lung parenchyma [7]. The capacity of oral dysbiosis to drive remote organ translocation via hematogenous pathways is further supported by recent neurological models; translational data demonstrate that the combination of advancing age and chronic periodontitis synergistically compromises structural vascular barriers, drastically increasing the systemic dissemination and brain tissue infiltration of oral bacteria [53]. When this same hematogenous vulnerability is applied to the respiratory system, the persistent influx of oral-derived inflammatory mediators, combined with a failed resolution response due to local SPM deficiencies [54], sustains an insidious inflammatory environment that compromises pulmonary compliance and accelerates chronic parenchymal damage. Pulmonary-specific translocation routes of oral pathobionts and their metabolites were schematized in Figure 2.

## 4. The Respiratory Impact: From Infections to Chronic Disease

The migration of oral microbes into the lower airways is more than a simple anatomical shift; it triggers a continuous immune response. During oral eubiosis, the lungs’ innate defenses effectively manage and clear routinely aspirated microbes. Oral dysbiosis exposes the lower airways to a steady stream of pro-inflammatory signals, altered metabolites, and keystone pathogens. This chronic microbial exposure acts as an underlying trigger for acute respiratory infections and the progression of chronic lung diseases.

### 4.1. The Diversity Deficit: From COPD to the Broader CRD Spectrum

The clinical impact of the oral–lung axis is particularly evident in Chronic Obstructive Pulmonary Disease (COPD) and other Chronic Respiratory Diseases (CRDs). While chronic airway obstruction has long been attributed to smoking, genetics, and environmental factors, it is now understood to be closely tied to microbiome shifts. Specifically, the progression from a healthy to a chronically inflamed respiratory tract is strongly associated with oral dysbiosis. Recent large-scale population studies have clarified this link, showing that a diverse oral microbiome is highly protective of lung health. For example, in large-scale human observational cohorts, a cross-sectional analysis of the NHANES database involving over 6000 US adults found a strong inverse relationship between oral microbiome diversity and the risk of COPD [55]. The data demonstrates a clear clinical difference: compared to healthy individuals, patients with COPD have significantly lower alpha-diversity metrics across multiple indices, including observed Amplicon Sequence Variants (ASVs), Faith’s Phylogenetic Diversity, and the Shannon-Weiner index [55]. Population-level data highlight the protective role of oral microbial richness: every 10-unit increase in observed ASVs corresponds to a 3.6% reduction in COPD risk [55]. The magnitude of this effect is notable: a 1-unit increase in the Shannon-Weiner index—reflecting both community richness and evenness—is associated with a 17.1% reduction in COPD risk. Similarly, a 1-unit increase in Faith’s Phylogenetic Diversity corresponds to a 4.5% decrease in risk [55]. This diversity deficit is not limited to COPD; it is a common feature across other Chronic Respiratory Diseases (CRDs), including asthma, emphysema, and chronic bronchitis [56]. A two-stage study combining NHANES data with an independent clinical cohort demonstrated that each standard deviation increase in observed ASVs and Faith’s Phylogenetic Diversity is associated with a 19% and 17% reduction in the overall risk of CRDs, respectively [56].

Caution is required when interpreting these associations, as observational epidemiological cohorts inherently cannot rule out unmeasured residual confounding—such as lifelong smoking history, socioeconomic factors, or concurrent medication use. Recent genetic causal inference models (Mendelian Randomization) have attempted to bridge this gap, yet conflicting evidence remains regarding whether specific pathobionts are true disease initiators or merely secondary colonizers of an already inflamed respiratory niche. Recent research has advanced our understanding of oral dysbiosis in respiratory disease, shifting the focus from observational associations to causal relationships. To evaluate potential causal directionality beyond observational correlation, human genetic causality models (bidirectional Mendelian Randomization) now indicate that specific oral microbial traits play a causal role in the pathogenesis of COPD [57,58]. Far from being mere opportunistic colonizers, taxa from genera such as *Fusobacterium*, *Prevotella*, and *Streptococcus* exert a direct causal influence on lung disease risk [57]. A recent large-scale genetic analysis involving East Asian cohorts has further clarified this causal relationship, identifying 18 bacterial species across the tongue dorsum and saliva that influence COPD susceptibility [58]. This genetic blueprint points to a dual role for the oral microbiome: opportunistic genera such as *Gemella*, *Actinomyces*, and *Aggregatibacter* possess traits that elevate COPD risk, while others, such as *Prevotella intermedia* and *Streptococcus mitis*, exhibit protective genetic signatures against airway obstruction [58]. The implications of this genetic causality extend beyond chronic diseases, providing valuable insight into the etiology of acute airway infections. A large-scale Mendelian randomization study has demonstrated that specific variations in the oral microbiome significantly influence host susceptibility to five distinct respiratory infections: tonsillitis, chronic sinusitis, bronchiectasis, bronchitis, and pneumonia [59]. This relationship is inherently bidirectional; the presence of COPD exerts a reverse causal effect on the oral cavity. The systemic inflammation associated with COPD alters the oral environment, favoring the proliferation of pathobionts such as *Rothia* and *Campylobacter*, while diminishing beneficial *Streptococcus* species, ultimately sustaining a chronic inflammatory feedback loop [57].

### 4.2. Mechanisms of Damage: Microbial Perpetrators and Inflammatory Triggers

The detrimental impact of reduced oral diversity on the lower airways stems from compromised colonization resistance. This dysbiotic state not only permits the overgrowth and subsequent aspiration of pathogenic flora into the respiratory tract but also drives systemic inflammation [55]. These translocated oral microbes exacerbate lung pathology by promoting the attachment of respiratory pathogens, stimulating excessive cytokine release, and driving the formation of recalcitrant biofilms [55]. Upon colonization, these pathogens secrete an Extracellular Polymeric Substance (EPS) matrix—a complex network of polysaccharides, proteins, and extracellular DNA (eDNA)—which physically protects them from immune clearance and limits antibiotic penetration [60]. Within this EPS matrix, pathogens utilize quorum sensing to coordinate virulence and differentiate into persister cells—metabolically dormant variants capable of surviving antibiotic concentrations orders of magnitude above the minimum inhibitory concentration (MIC), severely compromising standard antimicrobial therapies [60]. These biofilms are dynamic structures; through both active enzymatic dispersal and passive mechanical shedding, they continuously seed the lower airways, driving recurrent exacerbations and progressive tissue damage [60]. Before reaching the lower airways, these pathogens trigger a cascade of enzymatic degradation. Periodontal bacteria secrete specific degradative enzymes—such as sialidase, fucosidase, and mannosidase—that compromise the protective salivary pellicle and degrade the respiratory mucosal epithelium. This enzymatic activity unmasks previously hidden adhesion receptors, thereby facilitating the attachment of respiratory pathogens and predisposing the host to severe infections [61].

The altered respiratory microenvironment in COPD exerts a selective pressure that favors the proliferation of pathogenic bacteria over beneficial commensals [62]. This migration initiates a progressive cycle of chronic airway inflammation and tissue damage [62]. *Haemophilus influenzae* and other oral pathogens exhibit sophisticated immune evasion strategies, including the inhibition of macrophage apoptosis and the formation of persistent biofilms within the lung mucosa [62]. Following colonization, these pathogens induce the formation of Neutrophil Extracellular Traps (NETs) and stimulate the secretion of tissue-degrading proteases, such as MMP-12, leading to emphysema and impaired mucociliary clearance. The resulting dysfunction of the lung’s clearance mechanisms facilitates further aspiration and colonization by oral pathogens, exacerbating a continuous cycle of respiratory decline [62].

Beyond being a hallmark of stable COPD, reduced microbiome diversity serves as a dynamic contributor to disease progression. This loss of diversity is particularly evident in the frequent exacerbator phenotype, where oral and sputum dysbiosis are linked to increased AECOPD frequency [55]. Consequently, the dysbiotic oral cavity acts as a persistent reservoir, periodically introducing inflammatory triggers into the lungs that precipitate acute respiratory failure.

Specific taxonomic shifts within the dysbiotic oral cavity drive significant immune activation. As oral microbial balance shifts, there is a marked increase in pathobionts like *Rothia*, *Veillonella*, *Fusobacterium*, and *Leptotrichia* [56]. Upon entering the lower respiratory tract, these organisms stimulate the airway epithelium, triggering the excessive release of pro-inflammatory cytokines such as TNF-alpha, IL-1 beta, and IL-6 [56]. Evidence from genetic causality models corroborates this distal inflammatory stimulus, showing that pathogenic oral species drive the upregulation of systemic IL-1β, IL-6, and IL-17A [8]. The resulting systemic cytokine elevation triggers pathological signaling in the lungs, promoting a significant infiltration of neutrophils and macrophages that impairs the integrity of the lung epithelial barrier [58]. This persistent signaling facilitates chronic respiratory inflammation and subsequent tissue remodeling.

Current evidence indicates that the composition of aspirated oral bacteria may determine a patient’s specific inflammatory endotype. While *H. influenzae* colonization predominantly drives neutrophilic inflammation through CXCL8 activation, a different microbial signature involving *Granulicatella*, *Campylobacter*, and *Porphyromonas* is associated with eosinophilic COPD. Additionally, the ‘pauci-inflammatory’ endotype represents a distinct category in which inflammatory biomarkers are notably absent during exacerbations, potentially pointing to systemic comorbidities as the primary drivers. Identifying these microbial signatures provides a foundation for personalized therapeutic strategies [62,63].

The core of this destructive process lies in the breakdown of oral immune tolerance. In a healthy state, mucosal Langerhans cells (LCs) maintain homeostasis by releasing IL-10 and TGF-beta1, preventing the hyper-activation of effector T cells. As oral dysbiosis predominates, the mucosal surface is subjected to an increased load of PAMPs, leading to chronic activation of Toll-like receptors (TLRs). This persistent signaling shatters the delicate balance of immune tolerance, inducing a dysregulated cytokine response that impairs the epithelial barrier. This breach allows pathogens to penetrate the connective tissue and alveolar bone, establishing a primary route for systemic dissemination [64].

Genetic evidence highlights a distinct duality in the role of certain oral microbes across different respiratory diseases. For example, the genus *Prevotella* protects against pneumonia and tonsillitis but contributes to the pathogenesis of bronchitis. *Streptococcus* strains also demonstrate opposing roles; while *S. pneumoniae* is a key driver of pneumonia and tonsillitis, *S. pseudopneumoniae* is linked to a decreased risk of bronchitis. In contrast, Fusobacterium appears to be a consistent promoter of several conditions, including chronic sinusitis, bronchiectasis, and bronchitis [59].

Synergistic interactions between oral pathogens and established respiratory threats further complicate this microbial invasion. Keystone periodontal pathogens, including *Porphyromonas gingivalis* and *Fusobacterium nucleatum*, have been shown to potentiate the virulence of respiratory viruses (such as Influenza H1N1 and RSV) and lung-dwelling bacteria, such as Pseudomonas aeruginosa. These synergistic co-infections activate apoptotic pathways, such as the Bcl-2/Bax/caspase-3 pathway, in the respiratory epithelium, significantly increasing lung tissue injury and acting as a primary trigger for acute exacerbations (AE-COPD) [65].

Recent studies demonstrate that the periodontal ‘Red Complex’—consisting of *P. gingivalis*, *T. forsythia*, and *T. denticola*—is consistently present in both subgingival sites and tracheal aspirates during acute COPD exacerbations. This association is regulated via quorum sensing; Fusobacterium nucleatum utilizes AI-2 molecules to recruit *P. gingivalis* and *T. denticola*, forming the structural scaffold of these pathogenic biofilms. The proximity within the EPS matrix facilitates extensive Horizontal Gene Transfer (HGT), allowing for the rapid exchange of resistance genes and virulence factors via plasmids and membrane vesicles (MVs), which ultimately increases microbial virulence in the respiratory tract [61,66].

Beyond its role as a gateway, the oral cavity serves as a critical reservoir of antimicrobial resistance (AMR). Long before aspiration, oral pathogens undergo significant virulence acquisition within the biofilm environment. Metagenomic analyses indicate that *Streptococcus* species act as key genetic carriers of mobile elements, including the Tn916 transposase family, alongside macrolide resistance genes. This resistance transfer is further enhanced by the secretion of Membrane Vesicles (MVs and OMVs), which enable the dissemination of ARGs without cellular contact. These lipid-encased structures act as protective carriers, safely transporting plasmid DNA and virulence factors to the lower respiratory tract while shielding them from host exonuclease degradation [67]. This study indicates that bacteria within the biofilm matrix utilize extracellular nanotubes for the direct exchange of multidrug resistance genes, even between unrelated species. As the oral resistome develops from the first decade of life, microbes are already equipped with resistance mechanisms before reaching the lower respiratory tract or ICU environments. This early mobilization of genetic determinants within the oral cavity is a primary driver of antibiotic treatment failure in nosocomial pneumonia and acute exacerbations [67]. As the oral resistome develops from the first decade of life, microbes are already equipped with resistance mechanisms before reaching the lower respiratory tract or ICU environments. This early mobilization of genetic determinants within the oral cavity is a primary driver of antibiotic treatment failure in nosocomial pneumonia and acute exacerbations [68].

At the cellular level, in vitro cell culture assays demonstrate that *P. gingivalis* interferes with STAT3 cellular signaling in respiratory epithelial cells, exacerbating the apoptosis initiated by *P. aeruginosa*. The resulting synergy leads to greater cellular necrosis than in single-pathogen infections. To better understand these interactions, researchers are increasingly utilizing metatranscriptomics to capture the total RNA landscape. This ‘functional snapshot’ provides a more precise understanding of microbial activity compared to traditional DNA sequencing. To better understand these interactions, translational studies are increasingly utilizing metatranscriptomics to capture the total active microbial RNA landscape. While traditional DNA-based 16S sequencing confirms microbial presence, metatranscriptomic profiling serves as an exploratory, hypothesis-generating tool to identify transcriptionally active pathobionts (e.g., disproportionately active *H. influenzae* in COPD sputum). However, it is important to note that while metatranscriptomics offers profound mechanistic insights into host-microbe interactomes in experimental cohorts, its translation into routine clinical diagnostics remains experimental and constrained by high sequencing costs, rapid RNA degradation, and methodological non-standardization [61,69].

Beyond simple identification, recent evidence reveals that taxonomic presence does not always correlate with functional activity in the COPD lung. While 16S rRNA sequencing provides a taxonomic overview, metatranscriptome profiling distinguishes the transcriptionally active organisms truly involved in pathogenesis. Specifically, *H. influenzae* and *H. parainfluenzae* often demonstrate disproportionately high activity, indicating a dominant role in driving chronic inflammation and lung function loss. These findings necessitate a shift toward metatranscriptomic studies to capture the functional dynamics of the microbial-host interaction in COPD [70].

Dual RNA-sequencing (Dual RNA-seq) represents a significant advancement in studying the molecular dynamics of infection by simultaneously capturing the transcriptomes of both the pathogen and the host immune cell. This approach allows for the characterization of the interactive molecular cross-talk at the exact moment of infection. In studies involving human monocytes and pathogenic *Streptococci*, this simultaneous profiling has revealed how the host cell’s upregulation of cytokine cascades (e.g., CXCL8 and CXCL10) is met by the bacteria’s induction of immune evasion genes, such as sht, to resist phagocytosis and metal intoxication. Such dual-kingdom mapping offers a high-resolution view of immunopathogenesis, capturing the complex interplay within the respiratory interactome [71].

The respiratory virome, largely dominated by bacteriophages, plays a critical role in modulating the lung microbiota. These phages act as mobile genetic reservoirs that facilitate the transfer of virulence-related genes, driving the evolution of pathogenic characteristics and lung dysbiosis. This process follows a precise temporal sequence; Beyond bacterial communities, preliminary viral metagenomic investigations suggest a prospective phenomenon termed ‘lung virome convergence’. In exploratory critically ill intubated cohorts, a shift toward *Caudoviricetes*-dominated phages has been observed prior to nosocomial pneumonia onset, potentially modulating commensal abundance. However, these viromic and archaeal dynamics remain largely hypothesis-generating, requiring larger multi-center longitudinal studies to establish whether phage shifts are causal triggers or secondary biomarkers of host mucosal collapse [72,73]. Emerging translational models suggest that ‘trained immunity’—involving epigenetic and metabolic reprogramming of innate immune cells—represents a novel, potentially maladaptive axis in respiratory pathology. While ongoing exposure to oral microbial stimuli and DAMPs is hypothesized to induce histone modifications (e.g., increased H3K4me3 and H3K27ac) in alveolar macrophages, it must be emphasized that much of this mechanistic framework is derived from experimental models, and its precise clinical magnitude in human COPD cohorts warrants further prospective validation [74].

The pathogenesis of nosocomial respiratory failure involves a complex ‘inter-kingdom interactome’ that transcends bacterial communities. These ecosystems, comprising bacteria, archaea, viruses, and fungi, maintain homeostasis via metabolic and quorum-sensing pathways. During VAP or ARDS, this homeostasis is disrupted by the migration of gut-associated bacteria and yeasts, such as *Candida* spp., into the lungs. These fungal-bacterial associations form synergistic communities that enhance immune evasion and tissue invasion, often correlating with increased mortality. Additionally, the respiratory archaeome may modulate the metabolic environment, influencing gasotransmitter levels and pH gradients that select for virulent pathogens [75].

In addition to local damage, oral pathogens modulate systemic immune pathways. *P. gingivalis* and *A. actinomycetemcomitans* contribute to systemic autoimmunity through the citrullination of host proteins, facilitated by the PPAD enzyme and LtxA-mediated PAD hyperactivation, respectively. This process generates neoantigens that trigger ACPA production. The dysbiotic oral mucosa serves as a significant reservoir for these autoantibodies; before joint involvement, *P. gingivalis* stimulates NETosis via PAR-2 activation, leading to the release of citrullinated histones and local ACPA production. Additionally, *Prevotella* exacerbates this pro-inflammatory state by inducing aberrant glycosylation of circulating immunoglobulins, converting them into complement-fixing pathogenic agents [76].

In addition to local effects, *P. gingivalis* invades myeloid-derived dendritic cells (mDCs), stimulating CXCR4 expression without activating lymph node routing. This process enables the transport of pathogens to remote atheromatous plaques, contributing to cardiovascular disease progression. A significant synergistic effect occurs during co-infection with viruses like SARS-CoV-2. Periodontal pathogens release various proteases, such as gingipains and legumain, which can activate the viral Spike (S) protein and facilitate ACE2-independent entry into host cells. Additionally, these pathogens trigger the externalization of phosphatidylserine (PS) on host membranes. This apoptotic mimicry allows the virus-laden bacteria to be engulfed through TIM receptors, providing SARS-CoV-2 with a molecular pathway for invading tissues while bypassing immune surveillance [77,78].

The microbial imbalance observed in chronic lung disease is also reflected in the fungal kingdom. ITS sequencing indicates that severe pulmonary conditions trigger a marked expansion of Malassezia, suggesting that the mycobiome actively contributes to airway dysbiosis. Furthermore, recent multi-omics breakthroughs highlight the mycobiome as a critical component in the progression of lung decay. In vitro and preclinical experimental models indicate that species like *Candida* and *Aspergillus fumigatus* form integrated ‘trans-kingdom biofilms’ with *Pseudomonas aeruginosa*. Through quorum sensing, in vitro and preclinical evidence indicates that fungal-bacterial interactions (such as *Candida* spp. or *Aspergillus* co-cultured with *P. aeruginosa*) can form trans-kingdom biofilms with enhanced structural stability. While these synergistic inter-kingdom interplays offer compelling mechanistic models in vitro, their direct clinical impact on human airway resistance and therapeutic failure remains an active area of ongoing investigation [79,80].

Beyond bacterial interactions, the respiratory virome plays a critical role in maintaining airway homeostasis. Metagenomic sequencing indicates that a diverse bacteriophage population modulates the healthy lung microbiota. During virus-induced COPD exacerbations, however, this balance is disrupted, leading to a significant reduction in protective phage populations. The loss of this predatory pressure facilitates the unchecked overgrowth of opportunistic pathogens, which can fuel secondary bacterial infections [81].

**Figure 3:**
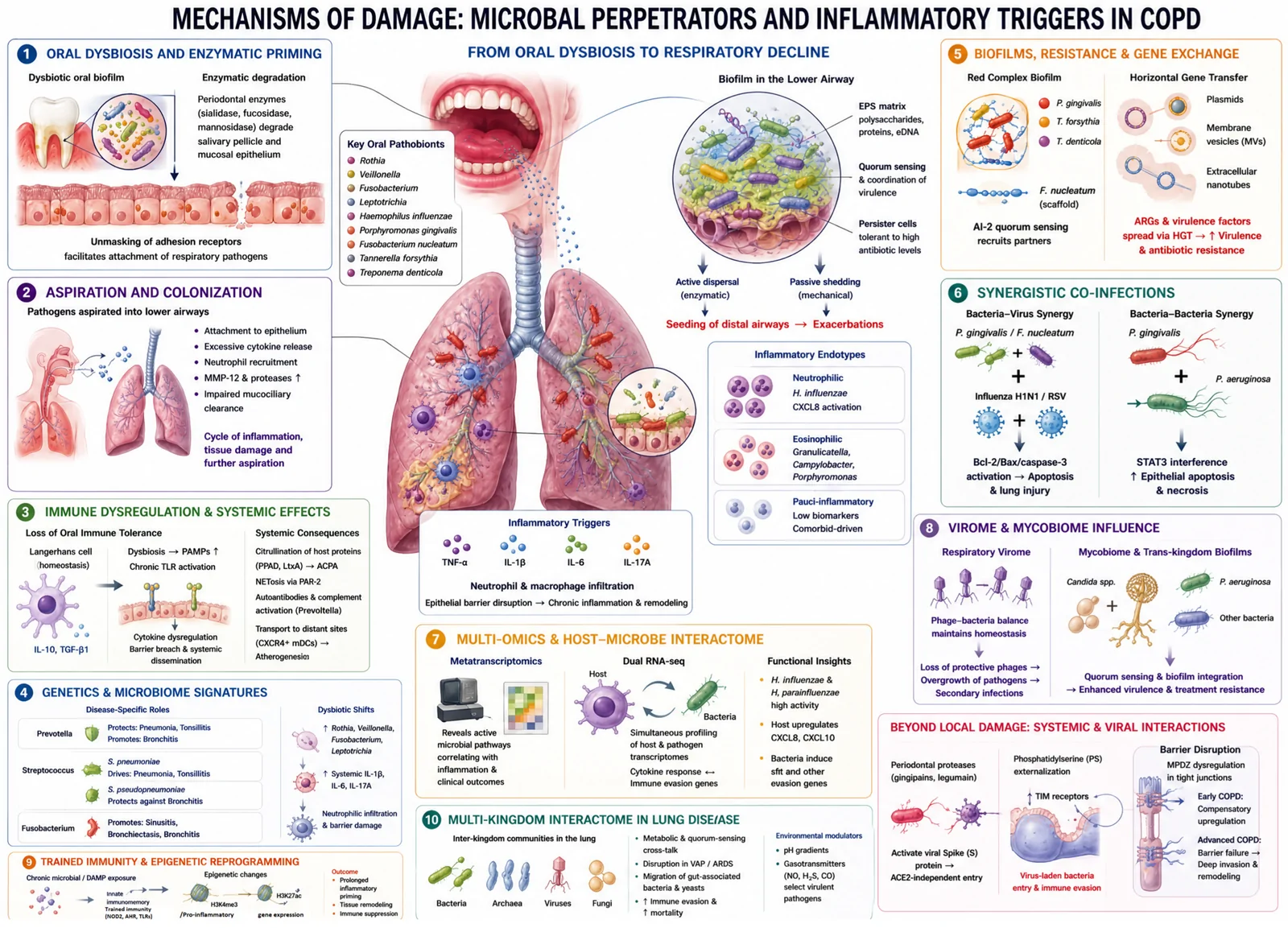
Comprehensive molecular mechanisms of damage, microbial perpetrators, and inflammatory triggers in COPD pathogenesis. Summarizes the multifaceted oral–lung interactome across 10 distinct thematic modules. Highlights oral enzymatic priming (sialidase, fucosidase unmasking adhesion receptors), biofilm matrix maturation (EPS scaffold, AI-2 quorum sensing, persister cell formation), horizontal gene transfer (plasmid and MV-mediated ARG spread), and synergistic co-infections *(P. gingivalis*/*P. aeruginosa* STAT3 inhibition; bacterial-viral apoptosis induction via Bcl-2/Bax/caspase-3). Further illustrates inflammatory endotypes (neutrophilic vs. eosinophilic vs. pauci-inflammatory), multi-omics profiling (metatranscriptomics and Dual RNA-seq), virome/mycobiome interactions (*Caudoviricetes* commensal lysis, *Candida*/*Pseudomonas* trans-kingdom biofilms), trained immunity (H3K4me3/H3K27ac epigenetic modifications), and tight junction barrier disruption via MPDZ dysregulation. Abbreviations: AI-2: Autoinducer-2; ARG: Antibiotic Resistance Gene; Bax: Bcl-2 Associated X Protein; Bcl-2: B-cell Lymphoma 2; COPD: Chronic Obstructive Pulmonary Disease; CXCL8: C-X-C Motif Chemokine Ligand 8; DAMP: Damage-Associated Molecular Pattern; EPS: Extracellular Polymeric Substance; HGT: Horizontal Gene Transfer; MMP-12: Matrix Metalloproteinase-12; MPDZ: Multiple PDZ Domain Protein; MV: Membrane Vesicle; NET: Neutrophil Extracellular Trap; PS: Phosphatidylserine; STAT3: Signal Transducer and Activator of Transcription 3; TIM: T-cell Immunoglobulin and Mucin-domain; TLR: Toll-Like Receptor.

Multi-omics and single-cell studies have characterized the molecular cross-talk between oral pathogens and the respiratory epithelium. The destruction of cell–cell junction assemblies and the impairment of the mucosal barrier represent key pathological events. Oral dysbiosis triggers the aberrant regulation of MPDZ, a hub gene essential for tight junction stability. In early COPD, MPDZ is upregulated in ciliated and endothelial cells as a compensatory defense against microbial challenges, alongside increased neutrophil infiltration. With disease progression and persistent microbial exposure, this barrier is ultimately compromised, leading to deeper bacterial invasion and chronic airway remodeling [57]. Mechanisms of microbial perpetrators and inflammatory triggers damaging the lung tissue were demonstrated in Figure 3.

### 4.3. The Loss of Commensal Shields: Metabolites and Immune Regulation

The pathogenesis along the oral–lung axis is driven not only by the invasion of pathogenic bacteria but also by a significant reduction in protective commensals. Health-associated genera, notably *Alloprevotella* and *Peptostreptococcus*, are markedly depleted in patients with chronic respiratory conditions. Additionally, emerging evidence indicates that genera such as *Pauljensenia* and *Capnocytophaga* function as protective commensals, with genetic associations suggesting a role in inhibiting the onset of pneumonia, bronchitis, and tonsillitis [56,59].

Commensal bacteria function as a critical barrier in mucosal immunity. *Alloprevotella* plays a key role in synthesizing short-chain fatty acids (SCFAs), such as acetate and succinate, which are vital for maintaining the respiratory barrier and regulating Th17-driven inflammation in asthma and COPD. Other commensals, such as *Peptostreptococcus*, maintain ecological balance through metabolic cross-feeding, preventing the proliferation of respiratory pathogens. When this protective microbial environment is disrupted by oral disease, the resulting loss of commensal stability compromises respiratory defenses, rendering the lungs vulnerable to infection and chronic disease progression [56]. Mucosal immunity involves competitive interactions between microbial species. *Streptococcus sanguinis* produces hydrogen peroxide (H_2_O_2_) as an inhibitor of pathogens such as *P. gingivalis*. This environmental pressure drives microbial adaptation, in which pathogens secrete KatA catalase to neutralize H_2_O_2_ and persist in the lung.

The loss of the commensals disrupts T-cell immune tolerance. While SCFAs normally support Treg differentiation and homeostasis, the loss of SCFA-producing species allows pathobionts to dominate the niche, favoring tissue-destructive Th17 polarization over protective Treg responses. This shift in the Th17 axis facilitates sustained mucosal inflammation and autoimmune processes [66,76].

The oral mucosa is protected by secretory Immunoglobulin A (sIgA), which plays a central role in maintaining homeostatic balance. Through immune exclusion, sIgA prevents the attachment of pathogenic species and fungi, such as *Candida albicans*, to the epithelial surface. This antibody is also essential for regulating the pathogenicity of oral flora and preserving alveolar bone structure. When oral dysbiosis disrupts this defensive network, the impaired ability to sequester pathogens enables colonization of local tissues and increases the risk of microbial aspiration into the respiratory tract [64].

### 4.4. The Vicious Cycle: Asthma and the Bidirectional Trap

The relationship between oral pathogens and pulmonary damage is bidirectional rather than unidirectional. Asthma exemplifies this interaction, in which chronic inflammation is further driven by microaspiration of *P. intermedia*. At the same time, respiratory therapies such as beta-2 agonists and inhaled corticosteroids can negatively affect oral health by reducing salivary flow and elevating glucose levels. These changes, combined with local immunosuppression, increase the risk of periodontal disease, dental caries, and oral candidiasis [82].

Fungal expansion contributes actively to pathogenesis rather than being a mere side effect of disease. *Candida* species produce significant quantities of prostaglandin E2 (PGE2), which promotes the alternative activation of alveolar macrophages into the M2 phenotype. This shift towards M2 polarization predisposes the respiratory environment to Type 2 immune responses, rendering patients more vulnerable to fungal-induced asthma attacks. Additionally, mycobiome profiling has pinpointed fungal genera that directly impair respiratory function. In asthma, the prevalence of *Cladosporium* and *Peniophora* correlates with reduced forced expiratory volume (FEV1), suggesting that fungal colonization contributes to airway constriction and functional decline [79,80].

**Figure 4:**
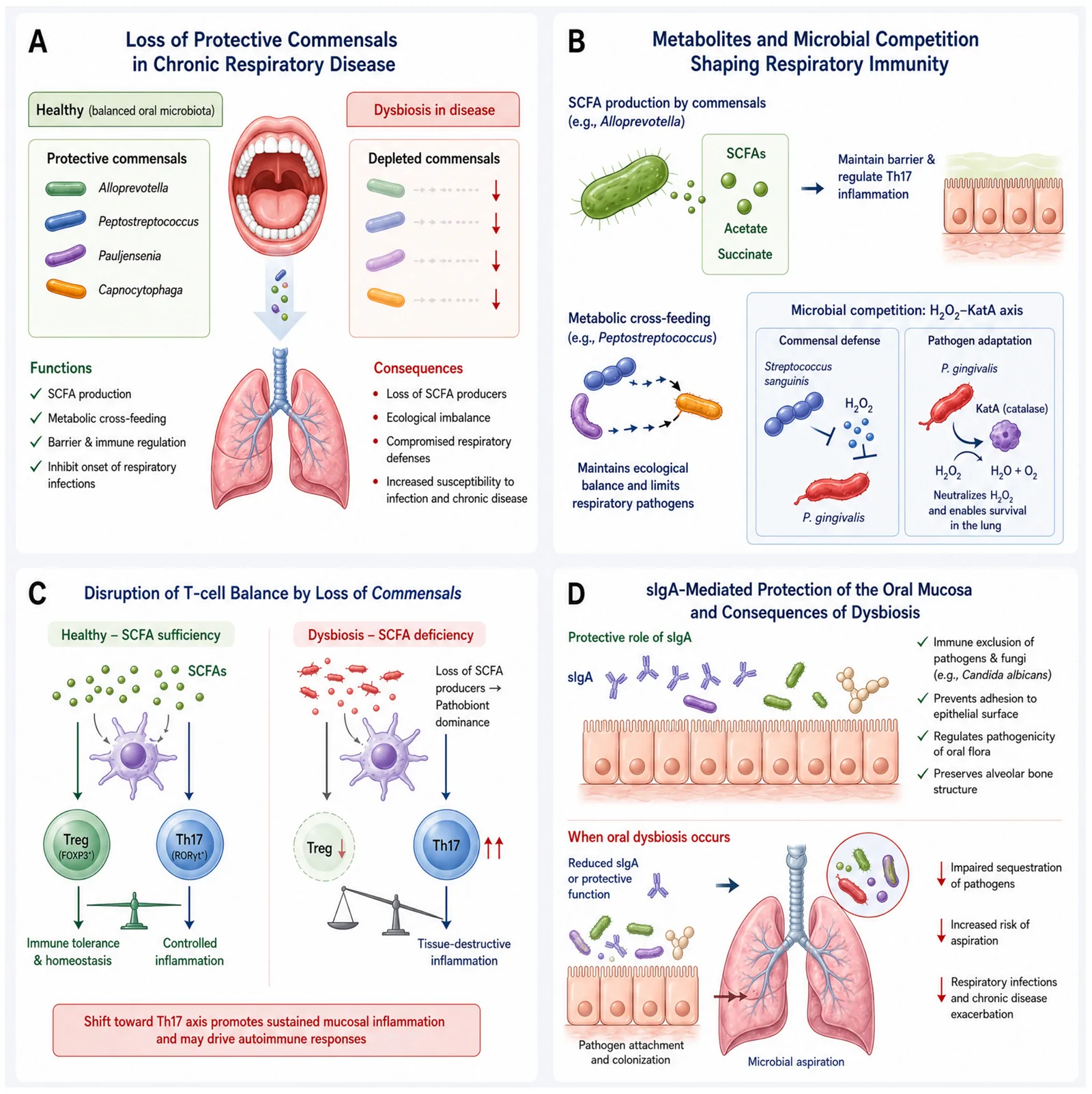
Immunological and metabolic consequences of protective oral commensal depletion. Panel (**A**): Depicts the shift from eubiosis to dysbiosis, showing the depletion of key protective commensals (*Alloprevotella, Peptostreptococcus, Pauljensenia, Capnocytophaga*). Panel (**B**): Explains commensal metabolic regulation, including short-chain fatty acid (SCFA) synthesis by *Alloprevotella* to support mucosal barriers, metabolic cross-feeding by *Peptostreptococcus*, and competitive microbial adaptation via the H_2_O_2_-KatA catalase axis between *S. sanguinis* and *P. gingivalis*. Panel (**C**): Illustrates T-cell tolerance disruption, where loss of SCFA-producing species shifts the Treg/Th17 immunological balance toward tissue-destructive Th17 polarization. Panel (**D**): Shows secretory IgA (sIgA) immune exclusion mechanisms, demonstrating how impaired sIgA sequestration allows pathobiont colonization and increases microbial aspiration risks. Abbreviations: H_2_O_2_: Hydrogen Peroxide; KatA: Catalase A; RORγt: Retinoic Acid Receptor-Related Orphan Receptor Gamma T; SCFA: Short-Chain Fatty Acid; sIgA: Secretory Immunoglobulin A; Th17: T-Helper 17 Cell; Treg: Regulatory T Cell.

The decline in oral health induced by respiratory medications contributes to a continuous influx of pathogens and inflammatory markers into the lungs, reinforcing a bidirectional cycle of disease. The virome further exacerbates this interaction; specifically, mNGS studies have highlighted the role of Rhinovirus C (RV-C), which shows a high affinity for the respiratory environment in asthmatic patients. By utilizing genetically upregulated receptors on the epithelium, RV-C can drive severe clinical exacerbations that characterize advanced asthma [81,82]. The consequences of the loss of protective commensal microbiota are outlined in Figure 4.

### 4.5. The Expanded Oral–Lung Axis: VAP, Aspiration Pneumonia, and Oncogenesis

Oral dysbiosis significantly increases the risk of acute respiratory infections, such as pneumonia. As the primary reservoir for these pathogens, the oral cavity contributes to the pathogenesis of community- and hospital-acquired pneumonia. In high-risk patients, the aspiration of oropharyngeal secretions facilitates the entry of oral anaerobes into the lungs. This process is driven by the unique environmental conditions of the subgingival pocket, which acts as a selective niche where anaerobes dominate aerobes by 1000:1. Species within the ‘Red’ and ‘Orange’ complexes are metabolically equipped for anaerobic survival. Upon reaching the hypoxic environment of diseased lungs, these pre-adapted bacteria find conditions suitable for colonization. Once established, they overcome initial host defenses, leading to clinical complications such as necrotizing pneumonia and purulent lung abscesses [36,82].

Mechanically ventilated patients are at increased risk of pulmonary infection because the ETT provides a direct route for pathogens to reach the lungs, bypassing mucosal barriers. Host protein films on the ETT surface enable multidrug-resistant species, such as *P. aeruginosa*, to establish persistent biofilms that continuously reseed the lungs, driving VAP. The long-held belief that the ETT cuff prevents the aspiration of oral secretions has been refuted by molecular analyses. 16S rRNA sequencing reveals a homogeneous distribution of microbial communities across the cuff, proving that these populations can migrate past the inflated seal to seed the lower airways. Despite these risks, the oral–lung axis offers a viable therapeutic window; professional oral hygiene interventions can significantly reduce bacterial burden, breaking the infection cycle and lowering the incidence of ventilator-associated and aspiration pneumonias [60,82,83].

The systemic impact of oral dysbiosis is increasingly linked to lung cancer etiology, even in non-smoking populations. Recent data indicate that a reduction in oral alpha-diversity and an abnormal prevalence of *Blastomonas* and *Sphingomonas* are associated with increased lung cancer risk. It is hypothesized that the chronic inflammation and immune evasion associated with periodontal disease promote a pro-tumorigenic microenvironment in the lungs. This cross-talk is mediated by systemic metabolic changes, in which lung cancer-derived exosomes travel to salivary glands to reprogram their gene expression. This process, influenced by MYC overexpression, leads to the abnormal secretion of acetylated polyamines, such as N1-acetylspermidine, into saliva, representing a novel aspect of the oral–lung oncogenic axis [82,84].

The relationship between fungal signatures and lung cancer is further highlighted by mycobiome mapping. Malassezia exhibits a significant increase in lung cancer patients, dominating the airway mycobiome, while Penicillium has been identified as a specific biomarker in this population. The role of *Malassezia* in carcinogenesis is mediated by its interaction with Mannose-Binding Lectin (MBL), triggering the complement cascade and fueling pro-tumorigenic inflammation. These shifts correlate with changes in the oral microbiomics, specifically a depletion in diversity and an overabundance of *Veillonella* and *Capnocytophaga*. Beyond the lungs, the oral pathogen *F. nucleatum* promotes gut cancer progression. By binding to Gal-GalNAc motifs on CRC cells through the Fap2 adhesin, *F. nucleatum* localizes within the tumor. It then interacts with TIGIT receptors on immune cells, facilitating evasion of host immune surveillance and driving tumor growth [77,79,85].

### 4.6. Systemic Crosstalk: The Gut–Lung–Oral Axis and Emerging Therapeutics

Respiratory health is inextricably linked to the systemic reach of oral microbes through the ‘gut–lung–oral axis’. Oral dysbiosis seeds the gastrointestinal tract with pathogens, leading to disruption of the intestinal microbiome and systemic inflammation that affects the lungs. This relationship is bidirectional: severe COPD exacerbations induce hypoxia, which increases intestinal permeability, allowing microbes and endotoxins to leak into the circulation. The foundation of this link is histological, as both tracts originate from the primordial foregut and maintain synchronized mucosal functions. This common origin facilitates a steady exchange through the blood and lymphatic systems. Consequently, intestinal dysbiosis may compromise the mucosal barrier, allowing toxins and allergens to reach the lungs and drive recurrent respiratory tract infections (RRTIs) [63,65,86].

Periodontal disease contributes to the systemic decline by disrupting the oral nitrate-nitrite-NO pathway. Commensal bacteria normally convert dietary nitrates into nitric oxide to support vascular and metabolic health. Dysbiosis in severe periodontitis significantly reduces these species, thereby lowering NO bioavailability, which impacts cardiovascular and immune function. These metabolic losses are as clinically significant as the introduction of pathogens. Consequently, new therapeutic opportunities are emerging; specifically, statins have demonstrated pleiotropic effects on the gut–lung–oral axis. By promoting the growth of anti-inflammatory species like *A. muciniphila* and *F. prausnitzii*, statin therapy can help reverse dysbiosis and mitigate systemic inflammation. This offers a promising strategy for stabilizing the respiratory microbiome and reducing COPD exacerbations [65,77].

Experimental therapies like Fecal Microbiome Transplantation (FMT) have demonstrated efficacy in slowing COPD progression by correcting the intestinal Firmicutes-to-Bacteroidetes ratio. This adjustment reduces systemic MMP-9 and MMP-12 levels, thereby limiting alveolar damage. Clinical translation of these findings has shown that multi-strain oral probiotics (e.g., Familact) significantly improve dyspnea in patients with GOLD stage III-IV COPD. While FEV1 levels often remain stable, patients report a statistically significant reduction in respiratory distress on the Borg and MMRC scales after two months of treatment. This highlights the potential of probiotics as adjunctive treatments to reduce the debilitating symptomatic burden of chronic respiratory conditions [62,87].

To provide a comprehensive synthesis of how oral dysbiosis intersects with specific pulmonary pathologies, the associated microorganisms, underlying pathological mechanisms, strength of evidence, and actionable clinical implications across the chronic respiratory disease spectrum are comparatively summarized in Table 1.

## 5. The “Aging” Factor: A Shifting Balance

Aging progressively disrupts the homeostasis of the oral–lung axis through interconnected biological changes [88]. This shifting balance is characterized by concurrent deterioration of structural barrier integrity, physiological reflex efficiency, and both local and systemic immune function, which increases susceptibility to respiratory complications stemming from oral dysbiosis [89,90].

### 5.1. Immunosenescence and the Escalation of Periodontitis

Central to this biological decline is immunosenescence—the progressive, age-associated remodeling of the immune system characterized by a diminished capacity to respond to novel antigenic challenges alongside a state of chronic, sterile, low-grade systemic inflammation known as “inflammaging” [91]. In the oral cavity, immunosenescence alters the leukocyte landscape within the periodontium; neutrophils exhibit impaired chemotaxis and reduced phagocytic efficiency, yet paradoxically release excessive amounts of tissue-destructive matrix metalloproteinases (MMPs) and reactive oxygen species (ROS) upon stimulation [7,92]. This dysregulated immune tone fails to control the subgingival pathobiont biomass while simultaneously driving collateral alveolar bone loss and mucosal tissue destruction [7,89]. Consequently, epidemiological and clinical data consistently show an age-dependent increase in the prevalence and severity of periodontitis, establishing the geriatric oral cavity as a persistent reservoir of virulent pathogens [93,94].

### 5.2. Breakdown of Physiological and Structural Barriers

Age-induced physiological and structural alterations successfully break the protective mechanisms of the shared aerodigestive tract:

- Salivary Hypofunction: Aging is frequently associated with acinar atrophy in the major salivary glands, leading to a qualitative and quantitative reduction in unstimulated salivary flow (xerostomia) [94]. The depletion of saliva compromises its vital flushing action and diminishes the concentration of essential antimicrobial proteins (such as secretory IgA and lysozyme), facilitating the uncontrolled colonization of opportunistic respiratory pathogens in the oral landscape [51,95].
- Neuromuscular and Reflex Decline: Senescent changes in the central nervous system and peripheral musculature lead to a progressive blunting of the pharyngeal swallowing and cough reflexes [90]. This neuromuscular impairment drastically increases the frequency and volume of silent micro-aspiration episodes, particularly during sleep, allowing toxic oral secretions to slip undetected into the lower respiratory tract [51].
- Impaired Mucociliary Clearance: Concurrently, the senescent pulmonary architecture exhibits a decline in ciliary beat frequency and altered viscoelastic properties of airway mucus, severely impeding the mucociliary escalator’s ability to clear the aspirated oral inoculum [96]. Together, these factors transform a highly regulated physiological pathway into a fragile and porous conduit for pulmonary infection.

### 5.3. Cellular Senescence and Intracellular Mechanisms

At the cellular level, the chronicity of periodontal tissue destruction in the elderly is driven by the accumulation of senescent structural cells [88,89]. Recent translational data have elucidated these precise intracellular dynamics using an innovative in vitro model in which primary human periodontal ligament fibroblasts (hPDLFs) were induced into a state of premature senescence via targeted D-galactose treatment [97]. This investigation demonstrated that D-galactose treatment significantly increases senescence-associated beta-galactosidase expression, oxidative stress, and inflammation while delaying wound closure and reducing cell viability and proliferation in hPDLFs, indicating acquisition of a senescence-associated secretory phenotype (SASP) [97]. Crucially, this structural failure can be successfully reversed; the application of specialized pro-resolving mediators (SPMs), specifically Resolvin E1 (RvE1) and Maresin 1 (MaR1), successfully restored senescent hPDLFs’ functional output, suppressing inflammation, restoring cell viability, and accelerating scratch-assay wound closure, with MaR1 demonstrating a more potent impact on reversing senescence than RvE1 [97]. This finding underscores that a deficiency in endogenous resolution pathways is a primary driver of age-associated oral-systemic breakdown.

### 5.4. In Vivo Evidence and Distant Organ Dissemination

The systemic gravity of this age-associated oral breakdown has been evaluated in preclinical in vivo animal models investigating the combined effects of advanced age and periodontitis. In preclinical murine models, experimental periodontitis drives maxillary bone loss and hematogenous dissemination of oral pathogens to distant organ tissues [53]. Preclinical evaluations demonstrated that aged murine models exhibit significantly greater maxillary bone loss in experimental periodontitis than their younger counterparts, accompanied by an altered inflammatory response and increased expression of senescence markers. Most remarkably, this research uncovered a definitive link between age-associated oral dysbiosis and distant organ infiltration via hematogenous routes: key periodontopathogens and oral commensals, including *Treponema denticola*, *Fusobacterium nucleatum*, *Porphyromonas gingivalis*, *P. pasteri*, and *Prevotella nigrescens*, were only detected in the brains of old animals with established periodontitis or were significantly more prevalent compared to young or periodontally healthy controls [53]. When translated directly to the oral–lung axis, these in vitro and in vivo findings provide a mechanistic basis for understanding how the combination of advanced age and chronic periodontitis not only drives a functional collapse of local tissue barriers but also actively fuels the systemic dissemination of oral pathogens and inflammatory mediators, ultimately predisposing elderly individuals to severe, non-resolving respiratory pathology and pulmonary decline [7,53,54].

## 6. Beyond the Lungs: The Multisite Interaction and the Mucosal Continuum

Beyond the oral–lung and gut-lung axes, the body operates through a ‘mucosal continuum’ where diverse microbial sites communicate with the respiratory system. The colonization of the infant airway—once thought to be sterile—is initiated by maternal vaginal and placental microbes during birth. This early microbial exposure is essential for establishing innate immune tolerance and determining future respiratory health. These interactions extend beyond simple translocation; microbial populations on the skin, gut, and oral cavity serve as systemic immune modulators. By releasing metabolites and PRR ligands into the circulation, these communities remotely influence bone marrow hematopoiesis. This process drives the differentiation of myeloid cells that populate the lungs, forming a critical component of the alveolar defense system [98].

The ‘gut–mycobiome–lung’ connection illustrates this complex inter-organ communication: an overgrowth of fungi such as *Candida* in the gastrointestinal tract can release circulating metabolites, including PGE2, which travel systemically to the lungs. These metabolites remotely modulate pulmonary macrophage polarization, exacerbating allergic airway inflammation. Within this mucosal continuum, immune equilibrium is regulated by the continuous activity of neutrophils and innate lymphoid cells, which modulate the IL-17/Th17 inflammatory axis to limit localized immunopathology. However, when prolonged oral dysbiosis leads to a profound disruption of local mucosal tolerance, the resulting immune dysregulation extends beyond the oral cavity [64,80].

The ‘skin–lung axis’ is demonstrated by the translocation of *Malassezia*. Although typically a skin commensal, this fungus can utilize the mucosal continuum to populate the respiratory tract during illness. That a skin-associated fungus becomes a major constituent of the lung mycobiome in conditions such as lung cancer further supports the porous nature of mucosal boundaries and indicates that they are systemically intertwined [79]. Long COVID (PASC) is increasingly linked to the role of the oral cavity as a persistent reservoir for SARS-CoV-2. The virus utilizes host proteins such as Neuropilin-1 and triggers ADAM17-mediated metalloproteinase shedding to exit the oral–lung axis, spreading to the central nervous system through vascular microthrombi. This persistent viral presence fuels chronic immune exhaustion, characterized by elevated iTreg and TGF-beta levels. Such systemic immune shifts are fundamental to the development of ‘brain fog,’ dysgeusia, and the fatigue seen in Long COVID patients [78]. Shifts in the vaginal, skin, or intestinal microbiota can trigger systemic responses that impact respiratory health. Such distal dysbiosis alters the lungs’ ability to clear aspirated oral pathogens and modulates the overall inflammatory environment within the respiratory tract [80,98].

The Female Reproductive Tract (FRT)–oral axis exemplifies the sex-specific nature of the mucosal continuum. The oral and reproductive systems share a bidirectional immunological relationship where oral dysbiosis-driven systemic inflammation increases the likelihood of bacterial vaginosis and alters salivary microbial profiles. Pathogens such as *P. gingivalis* and *F. nucleatum* are known to translocate from oral lesions via the bloodstream to the uterus and placenta, where they interfere with normal placental function. These microbial shifts are linked to serious pregnancy complications, including preeclampsia and preterm labor. Environmental stressors, such as smoking, further compromise this axis by depleting protective commensal populations, thereby strengthening the link between oral health decline and adverse reproductive outcomes [99]. The propagation of trained immunity to the bone marrow represents a critical level of systemic integration. Signals from oral or pulmonary dysbiosis reprogram hematopoietic stem and progenitor cells (HSPCs), driving a lineage bias toward pre-activated myeloid cells. Following their epigenetic modification, these cells infiltrate the respiratory mucosa as trained immune effectors, contributing to a systemic state of immunological priming. While this state may provide cross-protection against unrelated infections, it also risks amplifying the cytokine storm associated with severe viral pneumonia [74].

Beyond cellular defenses, the respiratory tract is characterized by a complex interaction between bacteriophages and the immune system. Phages are internalized by dendritic cells, leading to Toll-like receptor 3 (TLR3)-mediated interferon production that enhances antiviral responses. These viruses are also capable of directional transcytosis, enabling them to cross epithelial layers into the systemic circulation. While this interaction often supports host defense, an imbalanced virome can drive pro-inflammatory signaling via IL-1β and TNF. This process compromises pulmonary homeostasis and contributes to the progression of chronic diseases like cystic fibrosis [72].

The bone marrow acts as a central hub for this systemic continuum through ‘Maladaptive Myelopoiesis.’ LGSI, driven by oral dysbiosis, influences HSPCs through TRIM and CHIP. Epigenetic reprogramming via TRIM establishes a long-term ‘memory’ in the bone marrow, leading to sustained output of highly reactive myeloid cells. Concurrently, chronic inflammatory pressure drives CHIP, leading to mutations in genes like DNMT3A and TET2 and the subsequent clonal expansion of pro-inflammatory leukocytes. These genetically and epigenetically altered cells amplify systemic morbidity. This process is further exacerbated by the impairment of the vagus nerve’s cholinergic anti-inflammatory reflex, which limits the host’s ability to self-regulate systemic inflammatory responses [77].

## 7. Diagnostic, Preventive, and Therapeutic Horizons: Rewiring the Oral–Lung Axis

Given that oral dysbiosis underlies many cases of respiratory decline, the oral cavity offers a unique, non-invasive opportunity for early diagnosis and intervention. Acknowledging the mouth as a primary source of respiratory pathogens necessitates a shift in pulmonary management, emphasizing targeted prevention over purely reactive care [100].

The emergence of saliva as a non-invasive medium for monitoring respiratory health offers significant diagnostic potential. Saliva contains a diverse range of biomarkers, including proteins, metabolites, and cfDNA, enabling consistent assessment of pulmonary status. In COPD management, elevated salivary markers such as C-reactive protein (CRP), procalcitonin (PCT), and neutrophil elastase are used to predict acute exacerbations, while IL-8 and MMP-9 levels are associated with functional decline. Furthermore, saliva-based diagnostics are applicable in oncology; salivary cfDNA can identify key EGFR mutations, and microbial profiles involving *Capnocytophaga* and *Veillonella* provide a sensitive and specific means of detecting lung malignancies [85].

To improve diagnostic accuracy, it is essential to move beyond taxonomic identification and evaluate the active microbial community. RNA sequencing of sputum samples from COPD patients has identified that *Prevotella*, *Neisseria*, and *Fusobacterium* exhibit transcriptional activity that correlates with clinical severity. Distinguishing between the total microbiome (DNA) and the active metatranscriptome (RNA) enables identification of microbial drivers that govern disease progression. This distinction elucidates the biologically significant changes that characterize stable COPD and its acute exacerbations [70].

The expansion of salivary metabolomics, powered by advanced algorithms, has significant implications for respiratory diagnostics. Profiling the oral–lung axis reveals a 12-metabolite salivary signature, dominated by N1-acetylspermidine, that correlates strongly with the metabolic profile of lung tumors. This diagnostic strategy offers a precise, non-invasive means of screening for lung cancer regardless of demographic factors or smoking history. In the context of acute infections, saliva-based testing shows higher sensitivity for pathogen detection than nasopharyngeal swabs, while salivary viral load provides superior predictive value for patient outcomes [84,85].

Although saliva is a highly advantageous diagnostic medium, its clinical application has been hindered by the ultra-low concentrations of key biomarkers. Recent developments in nanotechnology have provided robust solutions to these challenges. The deployment of nanoparticles with specialized surface antibodies enables simultaneous enrichment and detection of low-abundance salivary components. Furthermore, integrating nanomicrofluidics and intraoral sensors enables real-time, point-of-care monitoring, significantly enhancing the diagnostic potential of the oral cavity in respiratory healthcare [101]. The detection of salivary pepsin serves as a key example of POC potential, identifying laryngopharyngeal reflux (LPR) as a driver of chronic airway irritation in asthma and COPD patients. Although liquid biomarkers offer significant diagnostic convenience, transitioning these microfluidic technologies from research to clinical practice poses significant hurdles [102]. The integration of radiomics with respiratory multi-omics represents a significant advancement in pulmonary diagnostics. Fusing quantitative CT-derived features with sputum microbiome profiles using methods such as WGCNA and wSNF enables detailed characterization of the lung interactome. By leveraging machine learning to integrate multidimensional datasets, researchers can overcome the constraints of single-omic analyses. This facilitates the identification of specific ‘pneumotypes,’ allowing for more accurate forecasting of lung function trends and the individualization of therapeutic strategies [69].

To be implemented in clinical practice, salivary and sputum biomarkers must meet stringent regulatory requirements. This multi-stage process includes analytical validation for precision, clinical validation for accuracy, and formal qualification by health agencies. These measures are critical for maintaining safety standards and preventing erroneous diagnoses that could compromise patient care in high-stakes environments [102].

### 7.1. Breaking the Chain in the ICU: Oral Hygiene as Pulmonary Shield

The oral–lung axis represents a critical target for prevention in intensive care settings. In critically ill and mechanically ventilated patients, oral dysbiosis promotes the accumulation of nosocomial pathogens. Implementing professional dental care in the ICU is recognized as a life-saving intervention rather than a routine hygiene measure. Evidence-based routines—incorporating brushing, calculus removal, and chlorhexidine—significantly lower the rates of LRTIs and VAP. Specifically, oral decontamination has been associated with a 56% reduction in the relative risk of LRTIs among ICU patients. Mechanical and chemical disruption of oral biofilms prevents the aspiration of pathogens into the lungs, thereby breaking the chain of hospital-acquired respiratory infections [100]. Managing artificial devices is a critical component of preventing respiratory decline.

Since endotracheal tubes and catheters act as significant reservoirs for microbial biofilms, current research focuses on advanced surface modifications such as zwitterionic polymers and silver nanoparticles. These coatings, along with enzyme-mediated approaches such as DNase I, are designed to prevent the initial stages of microbial colonization and to disrupt the EPS matrix, providing a proactive defense against Ventilator-Associated Pneumonia (VAP) [60]. Metagenomic Next-Generation Sequencing (mNGS) represents a significant advancement in respiratory diagnostics. While conventional PCR is restricted to specific panels, mNGS offers an unbiased blueprint of the entire respiratory microbiome. Culture-independent platforms such as Illumina MiSeq have demonstrated that ETT surfaces harbor far more complex microbial communities than those detected by traditional culture methods. These methods frequently uncover co-infections and opportunistic pathogens that often remain undetected in standard clinical settings. Given its high negative predictive value, mNGS is a reliable tool for excluding pathogens in critically ill patients with COPD. This comprehensive characterization of the microbial environment empowers physicians to refine antibiotic stewardship and protect the host’s indigenous microbiota [81,83]. The characterization of bacteriophage signatures offers significant potential for proactive respiratory care. Monitoring the endotracheal virome enables detection of a conserved profile comprising 66 bacteriophages, accurately identifying patients at risk for HAP before symptoms develop. Utilizing these markers enables early intervention and strategic modulation of the respiratory interactome, emphasizing the preemptive stabilization of the lung’s microbial equilibrium over traditional reactive treatments [73].

Using saliva to diagnose intracellular pathogens like Mycobacterium tuberculosis (Mtb) highlights several clinical limitations. Research shows that while individual salivary interleukins are not sufficient for a standalone diagnosis, they are effective tools for monitoring disease progression and the efficacy of Directly Observed Therapy (DOT) regimens. The clinical translation of salivary GeneXpert MTB/RIF assays is also hindered by the risk of aerosol generation during mechanical lysis, which necessitates BSL-3 laboratory conditions. Due to these biosafety requirements and cost constraints, these molecular platforms are increasingly viewed as community screening tools for initial identification rather than definitive confirmatory tests [103].

### 7.2. Vulnerable Populations: The Nasogastric Tube and Dysphagia Dilemma

Management strategies for elderly or neurologically compromised patients can inadvertently cause oral dysbiosis. Nasogastric (NG) tubes, by disrupting mechanical and salivary clearance, lead to a marked reduction in commensal *Streptococcus* and *Veillonella* while promoting the growth of pathogens like *Pseudomonas*. The resulting microbial profile closely resembles that of aspiration pneumonia, indicating that NG tubes prime the oral environment for respiratory complications. In patients with stroke or dysphagia, the impaired cough and swallowing reflexes facilitate the aspiration of these pathogen-rich biofilms, increasing the incidence of stroke-associated pneumonia (SAP). Implementing microbiome profiling of the tongue and saliva in these populations could serve as a predictive tool, enabling targeted antimicrobial strategies before the onset of clinical symptoms [100].

### 7.3. Dental Caries: From Tooth Decay to Pulmonary Predictor

Active dental caries is emerging as a clinical indicator of respiratory risk. High DMFT indices are no longer viewed solely as dental health metrics but as markers of pulmonary susceptibility. Patients with untreated decay are at increased risk for postoperative pneumonia following thoracic surgery. The acidic environment in carious lesions promotes the growth of opportunistic respiratory pathogens, such as *Veillonella* and *Prevotella*, which can subsequently seed the lower respiratory tract. Therefore, oral restoration and the re-establishment of a healthy microbiome are essential components of prehabilitation for patients with chronic lung conditions. Additionally, periodontal status is directly linked to respiratory function. Evidence shows that increased Clinical Attachment Loss (CAL) and the presence of subgingival *P. aeruginosa* are associated with a marked reduction in FEV1 and FEV1/FVC ratios [61]. Based on current clinical and genetic findings, integrating pulmonary evaluations into dental care and periodontal treatments into COPD management is becoming a necessity for interdisciplinary practice. Addressing oral health is particularly critical in maternal medicine; active caries and periodontitis are now recognized as markers for both respiratory complications and potential obstetric risks. Consequently, comprehensive oral care should be established as a fundamental part of prenatal healthcare protocols [58,99].

**Figure 5:**
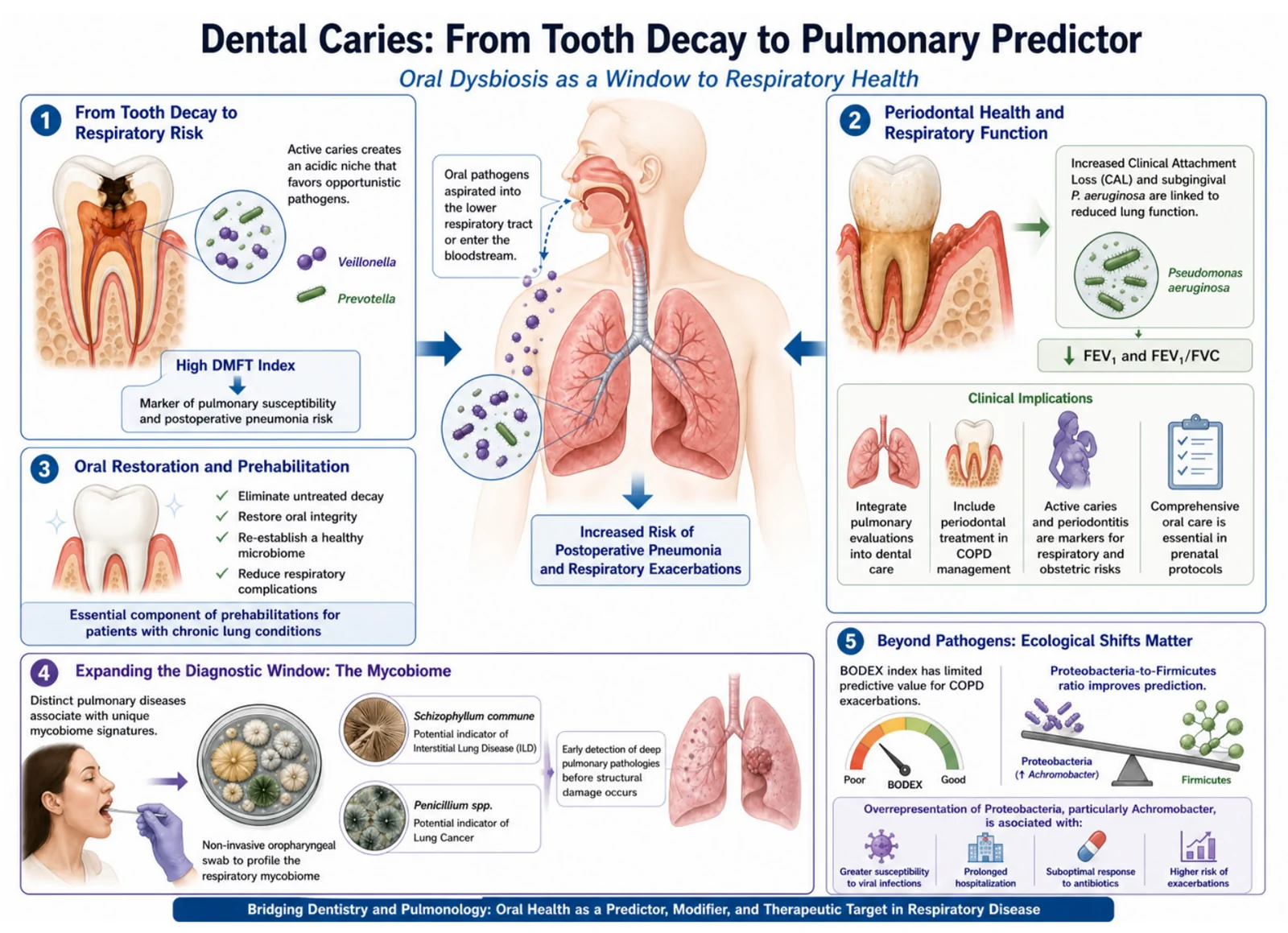
Dental caries and oral health metrics as clinical indicators of pulmonary disease susceptibility. Panel 1: Shows how active carious lesions (high DMFT index, acidic microenvironment pH < 5.5) enrich opportunistic respiratory pathogens (*Veillonella*, *Prevotella*), increasing postoperative pneumonia risk. Panel 2: Highlights the link between periodontal attachment loss (CAL), subgingival *P. aeruginosa* colonization, and reduced pulmonary function metrics (FEV_1_, FEV_1_/FVC). Panel 3: Outlines oral restoration prehabilitation strategies prior to thoracic surgery. Panel 4: Expands the diagnostic window to upper airway mycobiome profiling (*Schizophyllum* commune in ILD, *Penicillium* in lung cancer). Panel 5: Demonstrates ecological shift metrics, highlighting how an *elevated Proteobacteria*-to-*Firmicutes* ratio predicts viral infection susceptibility and antibiotic treatment failure. Abbreviations: BODEX: Body Mass Index, Airflow Obstruction, Dyspnea, Exacerbations Index; CAL: Clinical Attachment Loss; COPD: Chronic Obstructive Pulmonary Disease; DMFT: Decayed, Missing, Filled Teeth Index; FEV1: Forced Expiratory Volume in 1 Second; FVC: Forced Vital Capacity; ILD: Interstitial Lung Disease.

Moreover, this diagnostic framework now encompasses the fungal realm. Because distinct pulmonary diseases are associated with unique mycobiome profiles—such as Schizophyllum commune as a potential indicator of interstitial lung disease (ILD) and Penicillium as a potential indicator of lung cancer—non-invasive oropharyngeal swabs can be used to characterize the respiratory mycobiome. By analyzing these upper airway fungal signatures, clinicians can identify deep pulmonary pathologies at an early stage, potentially before significant structural damage occurs [79]. To enhance diagnostic accuracy, it is essential to move beyond pathogen identification and assess broader ecological shifts. Although indices such as BODEX have proved insufficient for predicting COPD exacerbations, measuring the Proteobacteria-to-Firmicutes ratio improves predictive accuracy. An overrepresentation of *Proteobacteria*, particularly *Achromobacter*, is associated with greater susceptibility to viral infections and is often linked to prolonged hospitalization and suboptimal responses to antibiotic therapy [63]. Figure 5 demonstrates dental caries as a clinical indicator of respiratory health risk.

### 7.4. Precision Interventions: From Targeted Mouthwashes to Intranasal Probiotics

The pharmacological management of respiratory diseases involves a complex balance between anti-inflammatory efficacy and mucosal integrity. Long-term administration of ICS or prophylactic azithromycin, while reducing acute episodes, has been associated with impaired mucosal defenses and a marked loss of microbial diversity in the airways. By inhibiting key molecules such as β-defensins, these treatments may predispose patients to secondary infections and facilitate the emergence of antibiotic-resistant strains [62]. Exposing respiratory biofilms to sub-MIC levels of antibiotics can act as an environmental stressor, complicating treatment. Rather than eliminating the infection, sub-lethal concentrations often promote biofilm development and facilitate the horizontal transfer of resistance plasmids among microbial populations [67].

To counteract iatrogenic dysbiosis, respiratory care is increasingly focusing on interventions that preserve and restore the microbiome. Advanced dental prophylaxis and targeted mouthwashes containing o-cymen-5-ol and zinc chloride have been shown to reduce the load of opportunistic pathogens, such as *Fusobacterium* and *Prevotella*, while maintaining commensal flora. This targeted approach is associated with a significant reduction in the frequency of COPD exacerbations [62]. Given that specific bacterial genera may exhibit antagonistic effects depending on the clinical context—such as inhibiting pneumonia while promoting bronchitis—indiscriminate microbial depletion is no longer a viable strategy. Modern approaches must instead prioritize precision in modulating the respiratory microbiome [59]. The intranasal delivery of *Lactobacillus* represents an emerging strategy for modulating the respiratory environment. Once established, these probiotics release metabolites, such as indole-3-acetic acid (IAA), that suppress neutrophilic inflammation. This biochemical pathway, mediated by macrophage-epithelial cross-talk, has been shown to slow the progression of structural lung damage and preserve respiratory function [62].

Oral probiotics contribute to respiratory health by regulating systemic immunity via the Gut-Associated Lymphoid Tissue (GALT). Interaction with M cells and dendritic cells in the GALT by strains like Bifidobacterium and Lactobacillus stimulates the formation of germinal centers and the expansion of IgA-producing B cells via TLR2 signaling pathways. These systemic interactions reinforce the mucosal immunity of the respiratory tract, providing broader protection against pulmonary infections [86]. High-quality clinical data support the role of systemic immunomodulation; oral probiotics containing Lactobacillus and Bifidobacterium species effectively modulate immune profiles by reducing pro-inflammatory cytokines (IL-1, IL-6, TNF-alpha) and increasing anti-inflammatory mediators (IL-10, IL-13). This polarization of T lymphocytes toward a Th1 phenotype represents a viable non-pharmacological strategy for addressing the chronic inflammatory milieu of the lungs, translating gut-mediated effects into clinical symptomatic improvement [87].

Advanced treatment strategies aim to disrupt the biochemical networks of respiratory biofilms. Quorum-sensing inhibition represents a viable method for destabilizing trans-kingdom microbial associations, making highly resistant pathogens more vulnerable to treatment. Novel approaches, including bacteriophage therapy and Zinc Oxide nanoparticles, are engineered to disrupt the structural integrity of the EPS matrix and eradicate persister cells. Biofilm stability can also be compromised by manipulating secondary messengers, such as cyclic-di-GMP, or by using enzymes such as Dispersin B to degrade structural polysaccharides. This induces active dispersion, thereby stripping pathogens of their protection and facilitating immune-mediated clearance.

Additionally, targeting the proteases involved in viral entry offers a promising antiviral strategy. Inhibiting microbial subtilisin, host ADAM17, and TIM-phosphatidylserine interactions can block the entry pathways used by viruses such as SARS-CoV-2, thereby reducing the formation of systemic reservoirs in the oral cavity [60,66,78,80]. Future therapeutic strategies for bone marrow reprogramming prioritize suppressing CHIP-mutant leukocyte expansion with mTOR inhibitors such as rapamycin. Advanced diagnostics now focus on identifying autoimmune conditions at the mucosal level. By monitoring sIgA autoantibodies and IgG glycosylation patterns, clinicians can detect high-risk profiles early in systemic autoimmunity, facilitating intervention before structural damage develops. Additionally, advancements in nanodentistry have introduced nano-theranostic devices that offer real-time monitoring of the oral cavity. These systems enable on-demand drug delivery as soon as dysbiosis is identified, thereby reducing the risk of microbial migration to the lungs [76,77,101].

### 7.5. Methodological Heterogeneity, Translational Gaps, and Causality vs. Association

To critically evaluate the oral–lung axis, published findings must be contextualized according to their inherent strength and level of scientific evidence. The current body of literature rests on a spectrum ranging from exploratory in vitro cell models to large-scale human genetic analyses. At the mechanistic baseline, in vitro and biofilm models—such as those examining enzymatic pellicle degradation, bacterial–viral STAT3 interference, extracellular polymeric substance (EPS) matrix formation, and trans-kingdom fungal–bacterial synergy—provide vital molecular proof-of-concept. However, these isolated cell culture environments lack host physiological clearance mechanisms and complex systemic immune architectures. Moving to preclinical in vivo frameworks, murine ligation-induced periodontitis and acute intratracheal pathogen instillation models demonstrate direct bacterial dissemination and pulmonary inflammatory responses. Nevertheless, these animal models rely on acute, high-dose bacterial challenges that fail to mirror the indolent, low-volume, decades-long kinetics of human micro-aspiration [49,51,53]. In human populations, large-scale observational cross-sectional and prospective cohorts (e.g., NHANES) confirm robust epidemiological correlations between oral health parameters—such as DMFT index or clinical attachment loss—and reduced pulmonary function or COPD prevalence [55,100]. While clinically highly relevant, observational studies cannot establish definitive temporality or completely rule out unmeasured residual confounding. To overcome these observational limitations, two-sample bidirectional Mendelian Randomization (MR) studies utilize human genetic variants as instrumental variables to infer potential causal relationships for specific oral taxa, such as *Fusobacterium* and *Prevotella* [57,58,59]. Although MR effectively circumvents reverse causation and traditional confounding, its conclusions remain constrained by potential horizontal pleiotropy, canalization, and population stratification.

A critical synthesis of the current clinical literature reveals several interconnected methodological, technical, and clinical limitations that necessitate cautious interpretation. First, substantial analytical heterogeneity stems from varying sampling modalities across studies. Supragingival plaque and non-stimulated saliva represent global oral shedding, subgingival pocket sampling captures localized anaerobic pathobionts, induced sputum evaluates central airway secretions, and invasive bronchoalveolar lavage (BAL) reflects distal alveolar microenvironments [14,22,51,70]. Crucially, lower respiratory specimens (particularly sputum and endotracheal aspirates) suffer from an inherent vulnerability to oropharyngeal cross-contamination during collection, which can artificially inflate the presence of oral taxa in pulmonary datasets [49,51]. Second, technical heterogeneity in microbiome sequencing platforms—ranging from variable 16S rRNA gene hypervariable region targeting (e.g., V1–V3 vs. V4) to shotgun metagenomics—introduces platform-dependent biases that hinder cross-cohort comparisons [69,70]. Third, clinical investigations are heavily confounded by major unmeasured or residual co-variables. Factors such as lifelong smoking history, recent broad-spectrum antibiotic usage, variable baseline oral hygiene practices, underlying systemic metabolic comorbidities (most notably type 2 diabetes), and biological age independently alter both mucosal microbial architecture and pulmonary host defenses [5,20,62,88,89]. Accounting for these critical confounders and sampling disparities is an essential prerequisite for future prospective, multi-center translational studies.

In summary, clinical validation varies significantly across concepts: while observational links between oral dysbiosis, periodontitis, and pneumonia risk are supported by robust epidemiological data, advanced frameworks such as respiratory virome convergence, trained immunity rewiring, trans-kingdom biofilm dynamics, and clinical metatranscriptomic profiling currently represent experimental, hypothesis-generating paradigms that require rigorous validation.

Methodological Limitations of Metagenomics and Metatranscriptomics in Respiratory Care.

While high-throughput metagenomic and metatranscriptomic sequencing platforms have revolutionized our understanding of the oral–lung axis, several technical and clinical constraints hinder their routine translational application. A fundamental limitation of DNA-based metagenomics is its inability to differentiate viable, metabolically active microorganisms from dead bacterial fragments or transiently inhaled non-colonizing DNA, which can lead to overestimation of pathogenic burdens. Additionally, lower respiratory tract specimens (e.g., sputum or bronchoalveolar lavage) often contain overwhelming amounts of host human DNA—frequently exceeding 95% of total extracted nucleic acids—requiring intensive host depletion steps that can alter true microbial ratios or introduce sample bias. Metatranscriptomics addresses functional activity by capturing active RNA expression, yet it introduces significant logistically demanding hurdles. Microbial messenger RNA (mRNA) possesses an exceptionally short half-life (often minutes) and degrades rapidly, demanding immediate flash-freezing or specialized RNA stabilization reagents at the point of collection, which is difficult to standardize in routine outpatient or ICU settings. Furthermore, both sequencing modalities suffer from high operational costs, lengthy turnaround times, variable 16S vs. shotgun sequencing depth, and a lack of internationally standardized bioinformatic pipelines and clinical cutoff values. Consequently, while these multi-omics platforms represent powerful exploratory tools for research, their diagnostic application in daily clinical pulmonology remains constrained [69,70,81].

### 7.6. Current Knowledge Gaps and Unanswered Questions

Despite significant progress in characterizing the oral–lung axis, several critical knowledge gaps remain to be addressed before basic findings can fully transition into standardized clinical practice. A fundamental unresolved question pertains to quantitative translocation thresholds; specifically, it remains unknown what precise bacterial load or subgingival pathobiont biomass (e.g., *P. gingivalis* or *F. nucleatum*) is required to breach local mucosal defenses and precipitate lower airway dysbiosis. Furthermore, the relative clinical contribution and precise temporal kinetics distinguishing continuous micro-aspiration from intermittent hematogenous translocation remain poorly defined across distinct clinical populations, such as mechanically ventilated ICU patients compared to ambulatory mild COPD cases [25,50].

Beyond the bacterial domain, while bacterial dysbiosis is well-documented, the functional dynamics of the respiratory and oral virome, mycobiome, and archaeome—as well as their complex multi-kingdom interactions—represent largely unexplored frontiers in mucosal immunology. From a therapeutic perspective, although professional oral care and decontamination protocols have been shown to reduce ventilator-associated pneumonia (VAP) incidence in critical care settings, large-scale prospective randomized controlled trials (RCTs) evaluating whether non-surgical periodontal therapy directly reduces COPD exacerbation rates, slows FEV_1_ decline, or improves all-cause survival in chronic respiratory disease cohorts are still lacking. Finally, regarding clinical translation, emerging salivary and sputum point-of-care diagnostics, such as nano-theranostic sensors and metatranscriptomic signatures, currently lack clinically validated diagnostic cut-offs and formal regulatory standardization necessary for routine pulmonology workflows [49,51,52].

### 7.7. Practical Clinical Applications and Interdisciplinary Care Frameworks

To bridge the gap between bench-side mucosal immunology and bed-side patient management, the findings of the oral–lung axis must be translated into actionable clinical pathways. Four practical domains represent immediate priorities for clinical integration:

1. Periodontal Care in COPD Management: Non-surgical periodontal therapy (NSPT), including professional mechanical scaling and root planing combined with targeted subgingival biofilm disruption, should be integrated into standard COPD care. Clinical trials demonstrate that treating underlying periodontitis significantly reduces circulating systemic inflammatory markers (e.g., high-sensitivity CRP, IL-6) and is associated with a lower annual frequency of acute exacerbations (AE-COPD) and improved quality of life metrics [36,61,62].
2. Standardized ICU Oral Hygiene Protocols: In critically ill and mechanically ventilated patients, oral decontamination represents a core life-saving intervention rather than a routine comfort measure. Standardized evidence-based protocols must incorporate gentle mechanical tooth brushing, subgingival calculus removal, continuous subglottic secretion suctioning, and chemical decontamination using 0.12–0.2% chlorhexidine gluconate or cetylpyridinium chloride. Such bundle approaches effectively disrupt pathogenic biofilm accumulation on endotracheal tube surfaces, lowering the relative risk of ventilator-associated pneumonia (VAP) by over 50% [60,100].
3. Interdisciplinary Pulmonology-Dentistry Collaboration: Effective management of the oral–lung axis necessitates a structured collaborative framework between dental professionals and pulmonologists. Pulmonologists should routinely screen chronic respiratory patients for oral health metrics (e.g., active caries, deep periodontal pockets, high DMFT index) and refer high-risk individuals for periodontal prehabilitation. Conversely, dental practitioners encountering severe periodontitis accompanied by chronic cough or dyspnea should prompt respiratory evaluations, such as spirometry screening [58,61,100].
4. Integration of Oral Microbiome Risk Scores into Respiratory Workflows: Assessing oral microbial dysbiosis via non-invasive saliva profiling or subgingival sampling offers a novel tool for respiratory risk stratification. Evaluating pathobiont abundance (e.g., Red Complex load or elevated *Proteobacteria*-to-*Firmicutes* ratios) can serve as a prehabilitative biomarker prior to major thoracic surgery, lung transplantation, or initiation of biological therapies, allowing clinicians to decontaminate the oral reservoir before invasive respiratory procedures [63,85,101].

### 7.8. Future Perspectives and Strategic Directions

To successfully translate the oral–lung axis from a compelling biological concept into routine clinical pulmonology and dental practice, future research must address key methodological gaps through prioritized strategic directions. First, future clinical investigations must move beyond cross-sectional designs to prospective, multi-center longitudinal studies. Tracking individual patients from early-stage respiratory disease through stable phases and acute exacerbation cycles—while combining continuous oral and lower airway multi-omics profiling—will be critical for determining true temporal sequences, identifying early dysbiotic biomarkers, and confirming causal directionality [55,69,70]. Second, to eliminate inter-study disparities, the international research community must establish standardized, accredited protocols for upper and lower airway specimen collection. This includes defining rigorous sampling techniques to minimize oropharyngeal cross-contamination, standardizing host DNA depletion methods, and harmonizing bioinformatic filtering pipelines for both 16S rRNA gene amplification and shotgun metagenomic or metatranscriptomic sequencing [49,51,69].

Furthermore, large-scale, well-powered Phase III interventional randomized controlled trials are urgently required to evaluate hard pulmonary endpoints. Specifically, future clinical trials should investigate whether incorporating professional non-surgical periodontal therapy, mechanical plaque disruption, or targeted oral probiotics into standard respiratory care directly reduces the annual frequency of acute COPD exacerbations, shortens the duration of ICU mechanical ventilation in VAP, or slows long-term decline in lung function [36,60,61,87]. Beyond the bacterial domain, future studies must prioritize the non-bacterial components of the mucosal continuum by investigating the functional dynamics of the oral and pulmonary virome, mycobiome, and archaeome to reveal novel inter-kingdom therapeutic targets [72,73,79,80]. Finally, translating oral biomarker discovery into clinical practice necessitates user-friendly, non-invasive diagnostic tools. Engineering efforts should focus on validating intraoral nano-sensors and microfluidic point-of-care devices capable of real-time detection of salivary inflammatory mediators and specific pathobiont abundance, enabling clinicians to intercept respiratory exacerbations prior to clinical manifestation [85,101,102].

## 8. Conclusions: A New Paradigm in Respiratory Care

Evidence regarding the oral–lung axis has significantly altered the traditional view of the ‘sterile lung.’ Modern medicine is transitioning toward an ecological perspective, where respiratory conditions are viewed as systemic mucosal imbalances rather than isolated airway failures. The respiratory tract functions as a dynamic ecosystem, home to diverse bacterial, viral, and fungal communities. The roles of fungal drivers such as *Malassezia* and *Cladosporium* highlight the importance of the mycobiome, establishing it as a central element in the pathogenesis of respiratory diseases [79,80,98].

The respiratory microbiome represents a dynamic ‘treatable trait’ rather than a mere consequence of disease. Current clinical practices are shifting away from indiscriminate use of broad-spectrum antibiotics, which can compromise essential commensal populations, and toward targeted microbiome engineering to preserve respiratory health [98].

Advanced clinical interventions are redefining the management of respiratory dysbiosis and immune homeostasis. These include host-targeted therapies, the targeted restoration of bacteriophage populations, and the use of inhaled probiotics or pharmabiotics. Recent developments also encompass nanoparticle-functionalized implants, protease inhibitors, and mTOR-based management of clonal hematopoiesis. Emerging tools such as smart nanotheranostics, salivary mass screening, and AI-driven metabolomic profiling facilitate early diagnosis. Furthermore, targeting the evolutionary hypoxic pre-adaptation of pathogens and utilizing targeted epigenetic editing or bacteriophage engineering provide novel pathways for treatment. The integration of high-resolution metatranscriptomic data and Dual RNA-seq host–pathogen mapping allows for a comprehensive understanding of real-time immunopathogenesis [36,60,63,69,70,71,72,74,75,77,78,81,84,86,98,99,101,103,104]. Given that physical barriers such as ETT cuffs cannot fully block microbial translocation, the future of intensive care requires a shift away from mechanical isolation toward targeted biological interventions for biofilm management [83].

The transition from viewing the respiratory system as a sterile compartment to recognizing it as a dynamic extension of the oral-systemic network marks one of the most significant paradigm shifts in contemporary medicine. As we move beyond the era of indiscriminate antimicrobial use, the oral cavity emerges not just as a gateway for pathogens, but as a sophisticated diagnostic mirror and a primary site for early intervention. The integration of advanced diagnostics—from salivary nano-sensors to high-resolution metatranscriptomics—now offers the potential to intercept pulmonary decline before it becomes irreversible.

Ultimately, addressing the complexities of the oral–lung axis necessitates a fundamental restructuring of clinical care. The successful management of chronic and acute respiratory diseases will increasingly depend on a collaborative framework in which pulmonologists, dentists, microbiologists, and immunologists work in a unified, multidisciplinary synergy. By embracing the principles of precision microbiome engineering and systemic immune modulation, we can move toward a future in which respiratory health is sustained through proactive stabilization of our indigenous microbial ecosystems, ensuring a more integrated and effective approach to human longevity and well-being.

## Figures and Tables

**Table 1:** Comparative summary of major respiratory diseases, associated oral microorganisms, underlying mechanisms, level of evidence, and clinical implications.

Respiratory Disease	Oral Microorganisms Involved	Proposed Mechanisms	Key Evidence/Strength of Evidence	Clinical Implications
COPD & AE-COPD	*P. gingivalis*, *T. denticola*, *T. forsythia* (Red Complex), *F. nucleatum*, *H. influenzae*, *Veillonella* spp., *Rothia* spp.	Micro-aspiration & hematogenous LPS leakage; STAT3 pathway interference; loss of SCFA-producing commensals (*Alloprevotella*); Th17 polarization; NETosis & MMP-12 activation.	Human observational cohorts (NHANES); Human genetic causality (Mendelian Randomization); Metatranscriptomics; In vitro/In vivo models.	Integration of non-surgical periodontal therapy into routine COPD care; monitoring alpha-diversity metrics.
HAP & VAP	*P. aeruginosa*, *S. aureus*, *P. gingivalis*, *Streptococcus* spp., *Caudoviricetes* phages, *Candida* spp.	Endotracheal tube (ETT) biofilm formation; loss of bacteriophage predator pressure (virome convergence 4–5 days prior); trans-kingdom fungal-bacterial EPS matrices.	Clinical prospective ICU cohorts; Viral metagenomics; ETT micro-sampling & 16S/mNGS profiling.	Implementation of ICU oral decontamination bundles (chlorhexidine, subglottic suctioning, mechanical brushing).
Aspiration Pneumonia & Abscess	Oral Anaerobes: *P. gingivalis*, *F. nucleatum*, *Prevotella intermedia*, *Peptostreptococcus* spp.	Massive micro-aspiration of toxic oral inoculum enriched in anaerobic pathobionts under hypoxic lower airway conditions; alveolar macrophage overload.	Preclinical animal models; Clinical microbiology of aspirates/abscesses; Epidemiological nursing home studies.	Oral hygiene prehabilitation in dysphagic/stroke patients; reduction in silent nighttime micro-aspiration.
Asthma	*P. intermedia*, *Cladosporium* spp., *Peniophora* spp., Rhinovirus C (RV-C)	Bidirectional trap: Inhaled corticosteroids/beta-2 agonists reduce salivary flow; microbial micro-aspiration fuels Type 2 immunity via fungal PGE2 production & M2 macrophage shift.	Human clinical cohorts; Mycobiome ITS sequencing; Pharmacological adverse-effect profiling.	Oral health monitoring in patients taking high-dose inhaled corticosteroids; intranasal/oral probiotic adjunctive care.
Respiratory Tract Infections (RRTIs, Sinusitis, Bronchitis)	*Streptococcus pneumoniae*, *S. pseudopneumoniae*, *Fusobacterium* spp., *Pauljensenia*, *Capnocytophaga*	Sialidase/fucosidase degradation of salivary pellicle unmasking hidden epithelial adhesion receptors; differential pathobiont/commensal susceptibility.	Human genetic causality (Two-sample East Asian Mendelian Randomization); In vitro enzymatic binding assays.	Targeted oral microbiome modulation rather than indiscriminate broad-spectrum antibiotic usage.
Lung Cancer (Oncogenesis)	*Blastomonas* spp., *Sphingomonas* spp., *Veillonella*, *Capnocytophaga*, *Malassezia* spp.	Chronic mucosal inflammation; complement cascade activation via fungal MBL binding; exosome-mediated reprogramming of salivary polyamines (N1-acetylspermidine).	Human salivary metabolomics; Tumor/Airway mycobiome profiling; Case–control clinical studies.	Non-invasive salivary metabolomic & cfDNA screening for early lung cancer detection and risk stratification.

## Data Availability

No new data were created or analyzed in this study. Data sharing is not applicable to this study.
